# Distribution, inducibility, and characteristics of *Latilactobacillus curvatus* temperate phages

**DOI:** 10.20517/mrr.2023.18

**Published:** 2023-09-08

**Authors:** Conrad L. Ambros, Matthias A. Ehrmann

**Affiliations:** Chair of Microbiology, School of Life Sciences, Technical University Munich (TUM), Freising 85354, Germany.

**Keywords:** *Latilactobacillus curvatus*, temperate bacteriophages, prophages, induction, lysis

## Abstract

**Aim:** Temperate phages are known to heavily impact the growth of their host, be it in a positive way, e.g., when beneficial genes are provided by the phage, or negatively when lysis occurs after prophage induction. This study provides an in-depth look into the distribution and variety of prophages in *Latilactobacillus curvatus* (*L. curvatus*). This species is found in a wide variety of ecological niches and is routinely used as a meat starter culture.

**Methods:** Fourty five *L. curvatus* genomes were screened for prophages. The intact predicted prophages and their chromosomal integration loci were described. Six *L. curvatus* lysogens were analysed for phage-mediated lysis post induction via UV light and/or mitomycin C. Their lysates were analysed for phage particles via viral DNA sequencing and transmission electron microscopy.

**Results:** Two hundred and six prophage sequences of any completeness were detected within *L. curvatus* genomes. The 50 as intact predicted prophages show high levels of genetic diversity on an intraspecies level with conserved regions mostly in the replication and head/tail gene clusters. Twelve chromosomal loci, mostly tRNA genes, were identified, where intact *L. curvatus* phages were integrated. The six analysed *L. curvatus* lysogens showed strain-dependent lysis in various degrees after induction, yet only four of their lysates appeared to contain fully assembled virions with the siphovirus morphotype.

**Conclusion:** Our data demonstrate that *L. curvatus* is a (pro)phage-susceptible species, harbouring multiple intact prophages and remnant sequences thereof. This knowledge provides a basis to study phage-host interaction influencing microbial communities in food fermentations.

## INTRODUCTION

Bacteriophages (short: phages, viruses specifically infecting bacterial cells) are known to negatively impact food production and cause considerable losses in revenue when the fermenting microbiota are inhibited by their antimicrobial activity, as seen in the dairy industry^[1]^. Nonetheless, phages can also be advantageously utilised, e.g., as additives to meat products to combat human pathogenic spoilage bacteria^[2,3]^, or as alternatives to antibiotics (phage therapy) in human health care^[4]^.

Phages are divided into virulent and temperate phages, depending on their replication behaviour. After infecting a bacterial host cell, virulent phages immediately hijack the translation and reproduction mechanisms of their host and new phage progeny (virions) are released after host lysis, which is caused by the interplay of different lysis proteins, such as holins and lysins (e.g., N-acetylmuramoyl-L-alanine amidases)^[5]^. In contrast, temperate phages can choose between the formerly described lytic life cycle, and the lysogenic life cycle, where the phage genome is stably integrated into the bacterial chromosome. The integrated phage genome is then called a prophage. In this life cycle, the prophage does not necessarily lyse its host and is replicated during cell division along with the host chromosome. The conversion from lysogenic into the lytic life cycle of temperate phages may occur spontaneously (spontaneous phage induction; SPI)^[6]^, but can also be forced by triggering the SOS response of the bacterial cell through DNA-damaging phage inducers like UV light, and various antibiotics such as mitomycin C^[7]^.

In contrast to the threat of lysogen depletion after their induction^[8]^, prophages can also positively affect their host’s fitness. Over the years, a multitude of different phage-derived mechanisms increasing host fitness have been discovered, such as the transduction of beneficial genes like antibiotic resistance genes (ARGs)^[9]^ to their host, or phage-encoded fitness factors like ADP-ribosyltransferases in *Bacteroides* that can enhance the colonisation capabilities of their host^[10]^. Furthermore, in *Lactococcus lactis*, the production of membrane vesicles, mediated by phage-related lysis genes and used as “decoy” by the host, has been discussed to reduce further phage infections^[11]^. In summary, an analysis of prophage distribution and the investigation of prophage-mediated functions can contribute to a deeper understanding of the competitive advantages of certain strains.

A wide variety of studies have shown that (pro)phages are commonly found in lactic acid bacteria (LAB)^[7,12-14]^. However, *L. curvatus*, a common starter organism in meat products serving as a bioprotective agent against spoilage bacteria^[15]^, has never been thoroughly screened for (pro)phages. *L. curvatus* is regularly consumed in the human diet, mainly in association with fermented meat products^[16]^. Moreover, it was isolated from kimchi^[17]^, sourdough^[18]^, dairy products^[19]^ and honey^[20]^, amongst others.

In a previous study, we examined the distribution and inducibility of prophages harboured within *L. sakei* and demonstrated that temperate phages are tightly connected to this species^[21]^. Considering that *L. curvatus* and *L. sakei* are phylogenetically closely related and share similar ecological niches, *L. curvatus* is a compelling potential host. Furthermore, recent publications evaluate the potential probiotic properties of *L. curvatus*^[17,22,23]^. This discussion can benefit from phage-related data, as phages have the potential to carry health-concerning genes such as toxins^[24]^ and are discussed to have a considerable impact on the gastrointestinal health of humans^[25]^.

## METHODS

### Bacterial cultivation

*L. curvatus* bacterial glycerol stocks (1:1,000 final dilution) were inoculated in fresh MRS broth^[26]^, in which ammonium citrate (mMRS) was substituted for di-ammonium hydrogen citrate (Carl Roth). The overnight cultures were incubated static at 30 °C with a closed lid until the next day.

### Prophage induction

For induction with UV light, overnight cultures were first diluted 1:50 in 50 mL fresh, preheated (30 °C) mMRS medium and incubated at 30 °C, static, with a closed lid, until an optical density (OD_600_) of 0.3-0.4 was reached. 10 mL of bacterial suspension was transferred into sterile 100 mL Erlenmeyer flasks and irradiated for 4 min with a UVT-28 M (Herolab) transilluminator, featuring eight 8 W UV-B light tubes (Herolab; Cat. No. 2984400; spectrum optimum at 312 nm wavelength). Homogeneous UV light exposure was ensured by shaking the cultures periodically during radiation treatment.

For mitomycin C induction, 15 mL preheated mMRS medium was set to an OD_600_ = 0.05 with overnight cultures. When an OD_600_ = 0.1 was reached, mitomycin C from Streptomyces (Sigma-Aldrich) was added in different concentrations (20, 10, 5, 0.5, 0.2, and 0 µg/mL).

Growth after induction was monitored at 30 °C in a FLUOstar Omega reader (BMG LABTECH GmbH) for 12 h. Measurements were taken every 5 min in a 48-well microplate format with 1 mL sample volume per well and double orbital shaking at 200 rpm prior to each measurement.

### Phage purification

Virions containing bacterial cell lysates were harvested 24 h after induction by sterile filtration using Filtropur S 0.45 µm filters (Sarstedt). Virions were precipitated by adding 4% (w/v) PEG 8000 (Sigma-Aldrich) and 0.5 M NaCl (Carl Roth) (both final concentrations) and incubating for 1 h on ice. After centrifugation (1.5 mL lysate volume: 4 °C; 13,000 × *g*; 10 min), the supernatant was discarded and phage pellets were solubilised in 1/10th of the former sample volume with SM buffer with gelatine^[27]^. For 1 L SM buffer with gelatine, 5.8 g NaCl (Carl Roth), and 2.0 g MgSO_4_·7 H_2_O (Sigma-Aldrich) were dissolved in 800 mL demineralised water and 50 mL 1 M Tris-Cl and 5 mL gelatine solution [aqueous solution with 2% w/v gelatine (Carl Roth)] were added before adjusting the volume to 1 L. For 1 L Tris-Cl, 121.1 g/L Tris base (MP Biomedicals) was dissolved in 800 mL water, and after adjusting the pH to 7.5 with concentrated HCl (Carl Roth), the volume was adjusted to 1 L with demineralised water. Sterilisation by autoclaving was performed at 121 °C for 15 min. Enriched phage samples were stored at 4 °C.

### Electron microscopy

Negative staining transmission electron microscopy (TEM) of enriched phage solutions was performed as described previously^[28]^. For this, 5 µL of the sample was applied to glow-discharged and carbon-coated copper grids. After blotting on filter paper, samples were washed twice with double-distilled water. After negative staining with 2% uranyl acetate for 20 s, samples were blotted again and air-dried. Electron micrographs were generated using a Zeiss EM912 (operated at 80 kV in the zero-loss mode) with an integrated Omega filter (Zeiss), utilising a 2 k × 2 k CCD camera (TRS).

### Cell disruption and DNA extraction

Bacterial genomic DNA was extracted from 0.8 to 1.5 mL of an overnight culture using the “E.Z.N.A.^®^ Bacterial DNA Kit” (Omega Bio-Tek) according to the manufacturer’s information. For cell lysis, 220 µL lysozyme solution [10 mg/mL lysozyme (Omega Bio-Tek)] solubilised in TE-buffer [1 mM EDTA-dihydrate (VWR™), 10 mM Tris (Gerbu), pH 8.0] was added to each centrifuged cell pellet, followed by incubation at 37 °C for 1.5 h. Additionally, cell disruption via glass beads was carried out using the FastPrep^®^-24 (20 s, 4 m/s, 24/2) from MP Biomedicals.

### Bacterial genome sequencing, assembly, and annotation

Extracted DNA was sequenced with the Illumina HiSeq technology by Eurofins (Germany). Unicycler version 0.4.8^[29]^ on usegalaxy.eu was used for contig assembly. Default parameters were used, and the FASTA file contig length cut-off was set to 1,000. The NCBI Prokaryotic Genome Annotation Pipeline (PGAP)^[30-32]^ was used for the annotation of the bacterial genomes.

### Viral DNA extraction and sequencing

For virion concentration, residual cell debris was removed from 10 mL virion-containing, post-induced (UV light) lysate by centrifugation (6,000 × *g*, 5 min, 20 °C) and sterile filtration (induction and filtration as described before). Virions were precipitated at 4 °C using 0.5 M NaCl and 10% (w/v) PEG8000 (final concentrations) until the next day. After centrifugation (16,000 × *g*, 30 min, 4 °C), precipitated virions were harvested by resuspending the phage-containing pellet in 500 µL SM buffer^[33]^.

Prior to viral capsid digestion, bacterial gDNA and RNA were removed by adding 1.25 µL DNase I (Qiagen) and 2.5 µL RNase A (10 mg/mL; Carl Roth) and incubating the samples at 37 °C for 1 h. Viral capsids were digested at 60 °C for 1 h after the addition of 1.25 µL Proteinase K (20 mg/mL; Omega Bio-tek), 25 µL 10% (w/v) sodium dodecyl sulfate (SDS; Serva) stock, and 20 µL 0.5 M EDTA (pH 8.0; VWR™). Samples were briefly cooled down to ambient temperature (app. 20 °C), then DNA was extracted by phenol-chloroform extraction and precipitated using ethanol (protocol after Center for Phage Technology^[34]^). Afterwards, DNA was dissolved in 30 µL Elution Buffer (E.Z.N.A.^®^ Bacterial DNA Kit; Omega Bio-Tek).

Sequencing of viral genomes was performed using Eurofins (Germany) INVIEW Virus Sequencing for dsDNA viruses. Assembly was performed as previously described for bacterial genomes, with the exception of using a 100 bp contig cut-off for the generation of FASTA files.

### Genome selection for PHASTER analysis

The genomes of 56 strains, including genomes at all assembly stages (“complete”, “genome”, “scaffold”, “contig”), were downloaded from the NCBI website (Last download: 10th August 2022). The first analysis with the JSpeciesWS Webtool (Ribocon GmbH; version 3.9.5)^[35]^ allowed the exclusion of genomes with high similarity (99.00 ANIb over an alignment percentage cut-off of 95%), ensuring a diverse genome set for subsequent analyses. The 16 strains excluded are listed in the supplemental material [Supplementary Table 1]. The 38 genomes remaining in the analyses are listed in the “Availability of data and materials” section. Furthermore, 7 strains from the in-house strain collection were also included in this study, and are now provided as well at the NCBI website. A total of 45 genomes remained for further analysis (“Availability of data and materials”), including the genomes provided within this study. Of note are two strains: Strain TMW 1.706, which shares high sequence similarities (100.00% ANIb over 99.95% of aligned nucleotides) with *L. curvatus* type strain DSM 20019^T^ and strain TMW 1.1447, which shares 99.52% ANIb over 95.39% of the aligned nucleotides with strain DRD-164. While we did not have access to the *L. curvatus* type strain for the induction experiments, strain TMW 1.706 serves as a closely related replacement. Strain DRD-164 was included as it seems to differ in its prophages, despite the overall high genome similarities to strain TMW 1.1447. Notably, *L. curvatus* strain VRA_2sq_f was included within this study, albeit its genome was found to have a low average nucleotide identity in relation to the genomes of all other included *L. curvatus* strains (e.g., 88.24% ANIb over 66.84% of aligned nucleotides with strain DSM 20019^T^). While this strain is listed as *L. curvatus* at the time of creating this study, and seems more closely related to this species than to other species within the *Latilactobacillus* genus (data not shown), this might change in the future.

### Prophage annotation

Detection, first annotation and completeness evaluation were performed with the web tool “Phage search tool enhanced release” (PHASTER)^[36,37]^. For the lysogenic *L. curvatus* strains with inducible prophages, PHASTER-derived annotations were additionally checked manually by BLASTing translated amino acid sequences against UniProt’s virus database (uniprot.org; BLASTp; E-Threshold: 10; Matrix: Auto; Filtering: None; Gapped: yes) and replaced whenever reasonable. Prophage visualisation and gene colour allocation were performed with the program SnapGene Viewer (Insightful Science; snapgene.com).

Isotype and anticodon prediction of phage tRNA genes (derived from NCBI annotation) were performed via tRNAscan-SE version 2.0^[38,39]^. Codon usage propensities were analysed via CLC Main Workbench version 8.1.4 (Qiagen). The secondary structure predictions derived from tRNAscan-SE 2.0 and CLC Main Workbench version 8.1.4 (Qiagen) were checked manually.

The nucleotide sequences of potential methyltransferase genes were BLASTed (BLASTN) against the REBASE nucleotide sequences database using default parameters (date: 23rd February 2023) at rebase.neb.com^[40]^ to verify a correct annotation and type of methylation.

Assigned (pro)phage names consist of their host strain name and a capitalised “p” for “(pro)phage”, as well as a number to distinguish different intact (pro)phages within one host. According to the “International Committee on Taxonomy of Viruses” (ICTV)^[41]^, all (pro)phage names were written in non-italic, even when including host identifiers (e.g., L. curvatus phage TMW 1.591 P1).

**Figure 1:**
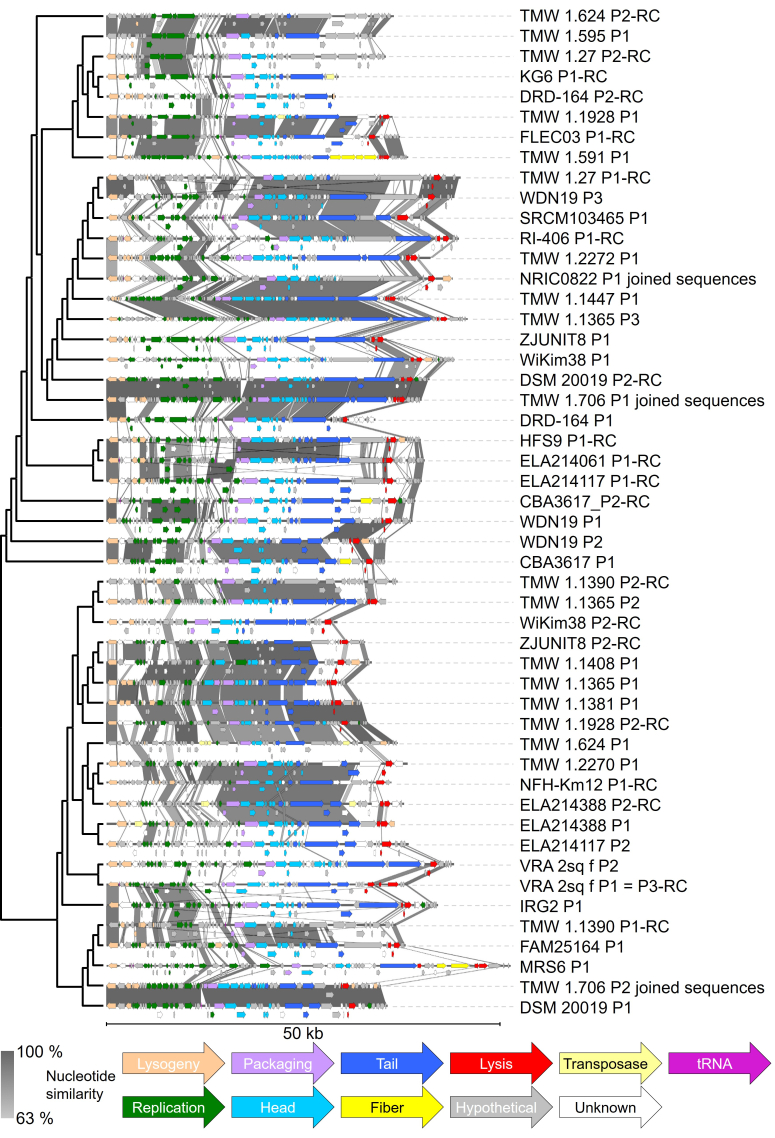
The 50 intact predicted *L. curvatus* prophages, pre-sorted by their phylogenetic relationship. Closer related phages are depicted next to each other. The phylogenetic tree is depicted as a cladogram and was constructed after whole genome alignment of those prophages**.** Prophages are displayed ranging from their predicted attL to their attR sites. Whole genome BLAST analysis was performed via Easyfig^[42]^ and indicates nucleotide similarities between 63% and 100% (darker bars between the sequences indicate higher similarity). “-RC” after the name of the phage indicates that the reverse complement sequence of the found phage region is displayed. Genes were colourised for their predicted task: Lysogeny (ochre), replication (green), packaging (lilac), head (light blue), tail (dark blue), fiber (yellow; includes host recognition genes), lysis (red), hypothetical protein (grey), transposase (light yellow), unknown task (white), and tRNA (pink). *L. curvatus*: *Latilactobacillus curvatus*.

The position of each prophage in Figure 1 is based on a whole genome alignment pairwise comparison (prophages with higher sequence similarity were depicted next to each other), using CLC Main Workbench version 8.1.4 (Qiagen). The phylogenetic tree in Figure 1 is depicted as a cladogram. Sequence similarity bars between each prophage are based on BLASTn, using Easyfig 2.2.5^[42]^ with the following settings: Min. length: 0; Max. e-value: 0.001; Min identity value: 0; Outline blast hits in black: yes; Filter small blast hits/annotations: yes. Genome alignment: Left side.

### Phylogenetic analysis

Using default settings, alignments were created with CLC Main Workbench version 8.1.4 (Qiagen). Construction of phylogenetic trees was performed using MEGA X version 10.2.6^[43]^ with the neighbor-joining method (distance measure: Maximum Composite Likelihood method). For the bootstrap analysis, using the Jukes-Cantor model, 1,000 replicates were used. All marker genes were extracted with the “extract annotation” feature of CLC Main Workbench and checked manually. The integrase outgroups used (lactobacilli phages infecting other species) are listed with their respective accession number in the supplemental material.

## RESULTS

### Prophage prediction in *L. curvatus*

To analyse the prophage incidence and distribution within *L. curvatus*, 45 genomes were searched for prophages with PHASTER (phaster.ca)^[36,37]^ and all identified phage-related sequences were classified by it as intact, questionable, or incomplete [Supplementary Table 2]. 206 sequences were detected in total; 47 were classified as intact (with zero to three sequences per strain), 38 as questionable (zero to six sequences per strain) and 121 (zero to eleven sequences per strain) as incomplete. All strains harboured prophages of some completeness level, and there were no strains with zero detected phage-related sequences. The number of phage-related sequences per strain of any completeness level ranged from one to 14. In a similar fashion to our previous prophage screening in *L. sakei*, PHASTER struggled to detect the exact borders of some of the intact *L. curvatus* prophages and occasionally misinterpreted one intact prophage as two sequences (e.g., as an intact prophage with an overlapping incomplete one, like in *L. curvatus* strain TMW 1.2272). Furthermore, bacterial genome regions with a high density of transposases were sometimes misjudged as (intact) prophages (e.g., in *L. curvatus* strain TMW 1.411).

To avoid these oversights, we manually checked the presence of all essential gene modules and determined attachment sites (att-sites) by aligning the outer regions of each prophage (including a bacterial genome part of a view thousand base pairs) onto one another. The att-site near the phage integrase was annotated as “attL”, and near the lysin as “attR”. Mostly, attL and attR are identical, or almost identical, short (10-25 bp) nucleotide sequences, which indicate the boundaries of the prophage and therefore mark its exact integration locus. Incomplete prophages often lack genome parts (including att-sites), so assigning an exact integration locus is difficult and inaccurate. With this in mind, only the integration sites of putatively intact prophages were analysed [Supplementary Table 3].

Following manual evaluation, 50 putatively intact *L. curvatus* prophages were found across 33 strains (intact prophage incidence of 73% in 45 analysed genomes) after manual evaluation. Notably, this includes transposase containing prophages, described below, and phages with only partially sequenced genomes. This revealed multiple putatively intact prophages in strains where no intact prophages were previously detected by PHASTER (e.g., *L. curvatus* strain ZJUNIT8). The identified prophages start (attL site) with lysogeny-related genes, followed by genes for replication, packaging, head and tail construction, including fibre and receptor genes, and lysis genes at the end (attR site). The integration locus within the host chromosome and the predicted att-sites for each intact predicted prophage are listed in Supplementary Table 3. General features like length, number of coding regions (CDS), GC content, and number of tRNA genes present in each prophage (including partial tRNA genes used for integration) are listed in Supplementary Table 4.

To display the genetic diversity of those prophages, we pre-sorted them based on a neighbour joining tree (Figure 1 left side; depicted as cladogram), which was constructed using a pairwise comparison table [Supplementary Figure 1], originating from a whole genome alignment using the phage genomes (attL to attR). The BLAST analysis revealed higher nucleotide similarities between closer related prophages, mostly in the replication and tail gene modules (compare Figure 1; grey bars). Notably, the two phage pairs TMW 1.706 P1/DSM 20019 P2 (96.31% nucleotide similarity over 100.00% aligned nucleotides) and TMW 1.706 P2/DSM 20019 P1 (97.69% nucleotide similarity over 100.00% aligned nucleotides) are almost identical. For this analysis, the split genome parts of prophages TMW 1.706 P1, TMW 1.706 P2 and NRIC0822 P1 have been joined. A correlation between phylogenetic groups of the bacterial hosts [Supplementary Figure 2] and the grouping of their respective prophages [Figure 1] could not be detected.

The shortest complete prophage detected was phage WiKim38 P2, with a length of 29.3 kb. Phage DRD-164 P2 was slightly shorter with 29.1 kb, but lacked lysis genes and could therefore be incomplete. Nevertheless, we included it in our analyses, as it contained all other gene modules and therefore relevant sequence information. The longest prophage was phage MRS6 P1 with 51.0 kb. The average intact prophage size was 37.8 ± 4.8 kb (median ± interquartile range). The GC content of the detected prophages ranged from 37.9% to 43.5%. The number of CDS ranged from 40 (phage KG6 P1, and phage TMW 1.595 P1) to 71 (phage MRS6 P1, and phage TMW 1.1365 P3). Each prophage harboured between 0 and 2 tRNA genes, not counting partial tRNA-sequences used for integration [Supplementary Table 4].

Of the 50 intact predicted prophages, 33 prophages harboured no tRNA genes, 13 included one tRNA gene, and 4 included two tRNA genes. Predicted tRNAs extracted from the NCBI annotation most often coded for isoleucine (9x) and phenylalanine (7x), but tRNAs for histidine (1x), and methionine (1x), as well as tRNAs with non-assignable isotype (3x) were also predicted.

The isotype prediction of the formerly undefined tRNA genes failed because of an erroneous secondary structure prediction (clover leaf form). The gene formerly classified as methionine tRNA was predicted as an isoleucine tRNA with CAU as anticodon (Ile2) by tRNAscan-SE 2.0. Including the formerly methionine tRNA classified tRNA, seven of the now ten predicted isoleucine tRNA genes were found to use CAU as an anticodon, one used AAU, one UAU, and one GAU.

Comparing the codon usage of the eight prophages harbouring isoleucine tRNAs with the codon usage of their host (including the prophage) revealed that, of the three codons for isoleucine (ATA, ATC, ATT), the eight phages used ATA (15.68 +/- 2.51)% (mean +/- standard deviation) the least, followed by ATC (24.58 +/- 2.35)%, and ATT (59.74 +/- 3.19)%. The eight host genomes used ATA (4.31 +/- 0.62)% for isoleucine coding the least as well, followed by ATC (31.08 +/- 1.47)%, and ATT (64.61 +/- 0.92)%. Compared to a phage harbouring no tRNA genes, e.g., phage TMW 1.591 P1, a similar distribution was observable (phage TMW 1.591 P1: ATA 11.54%, ATC 30.34%, ATT 58.12%; host strain *L. curvatus* TMW 1.591: ATA 3.65%, ATC 31.99%, ATT 64.36%). The analysed phages seem to use the ATA codon more frequently than their host in relation to the other isoleucine codons.

In contrast to this, the differences between host and phage in the usage of the two phenylalanine codons TTC and TTT were less pronounced. The phenylalanine tRNA harbouring prophages preferred TTT (63.65 +/- 3.41)% over TTC (36.35 +/- 3.41)%, as did the hosts at a similar rate [TTC was used by their hosts (41.41 +/- 0.51)% of the time, TTT (58.59 +/- 0.51)%]. Although TTC was used slightly less frequently by the phages relative to both phenylalanine codons, phenylalanine tRNA genes harboured by intact prophages used GAA exclusively as anticodon.

Five putatively intact prophages contained transposases. Four of them contained transposases, which belong to the IS3 and IS30 families. Both putatively intact prophages in *L. curvatus* strain ELA214388 shared IS3 family transposases: ELA214388 P1 in the lysogeny gene module and ELA214388 P2 in the replication gene module. ELA214388 P2 harboured an additional IS30 family transposase in its tail gene module. Phage TMW 1.1928 P1 contained an IS3-like element (IS1520 family transposase) in the head gene module and phage KG6 P1 contained an IS30 family transposase in its tail genes module, which may “cut off” the lysis genes, as a potential attR-site was found between transposase and lysis genes. Phage TMW 1.624 P1 contained four transposases lacking family affiliation according to annotation; three transposases were located in the replication gene module, and one in the tail gene module. After sequencing the post-induced strain lysates to verify successful prophage induction, the genome of phage TMW 1.1365 P2 also showed a gene with a putative transposase activity. This region was located at the ends of a contig within the NGS-sequenced bacterial genome, and was previously not allocated to the prophage.

Notably, in partially sequenced genomes with an assembly status of “contig” or “scaffold”, some putatively intact prophages also lacked features located at their boundaries, such as an att-site, or lysogeny or lysis-related genes, due to incomplete sequencing. For example, phage TMW 1.1447 P1 lacked most of its lysogeny gene cluster; phages TMW 1.706 P1 and TMW 1.706 P2 both contained non-sequenced parts in their replication genes; phage NRIC0822 P1 was harboured by two small contigs with most likely missing sequence information, as well as a non-determinable attR-site; phage TMW 1.1365 P2 had a non-determinable attR-site; phage VRA_2sq_f P1 = P3 had both att-sites sequenced, hinting at integration into a tRNA gene for serine, yet no tRNA gene was annotated as the corresponding sequence information was missing.

Methyltransferase genes were annotated in the replication gene modules of sixteen intact predicted prophages [Supplementary Table 5], with one to two of those genes per prophage. Of the 18 annotated methyltransferases, seven were marked as adenine-specific, eight as cytosine-specific, and for three, no specificity was determinable (checked via BLAST at uniprot.org). In direct proximity, only three prophages (phage FAM25164 P1, phage MRS6 P1, and phage TMW 1.591 P1) harboured downstream genes coding for a product with a predicted endonuclease function (tyrosine recombinase, or HNH homing endonuclease).

**Figure 2:**
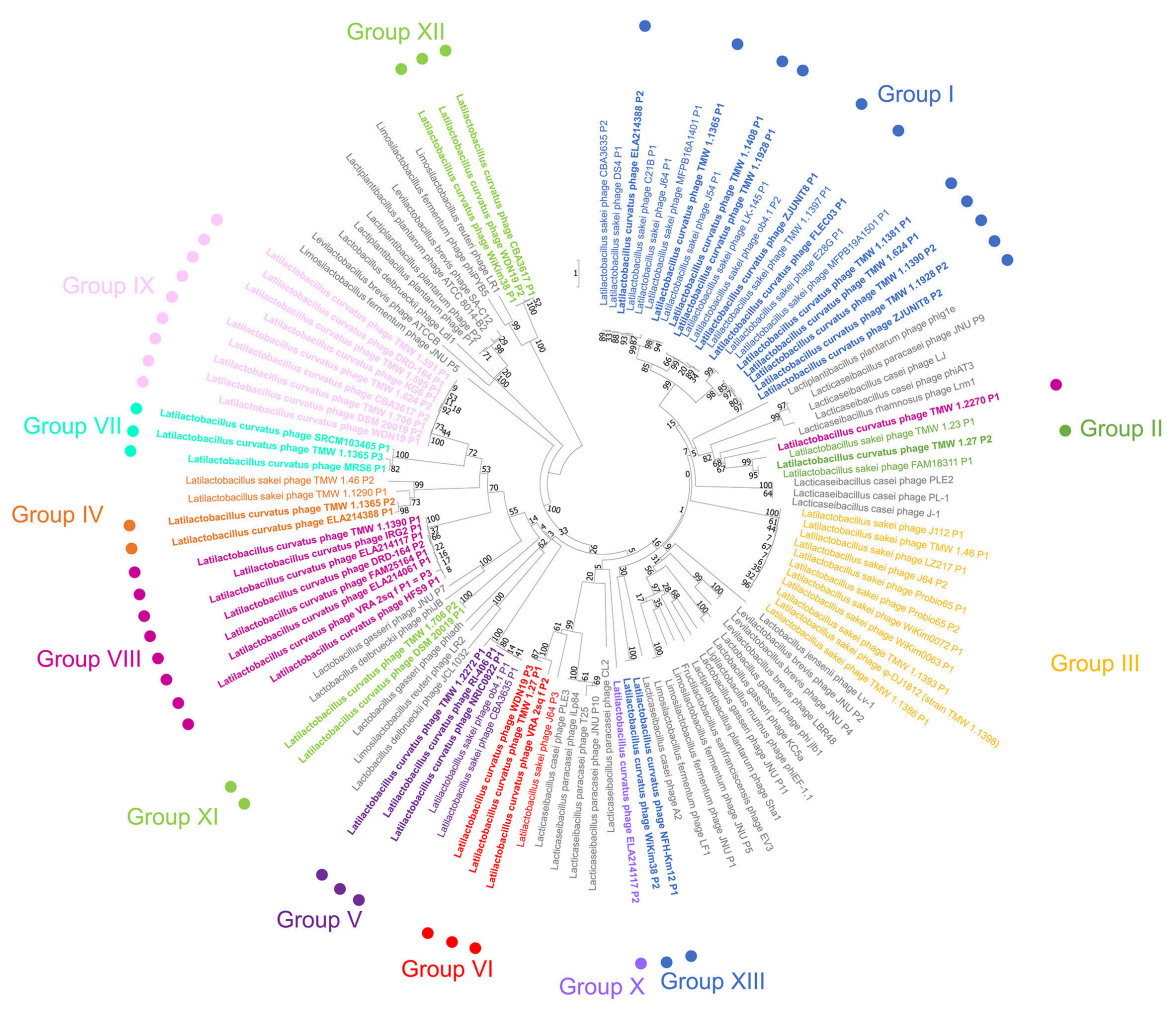
Phylogenetic tree after the neighbor-joining method (distance measure: Maximum Composite Likelihood method) of *L. curvatus* phage integrases. Phages infecting other lactobacilli were included as outgroups (grey lettering). The assignment of groups corresponding to closely related integrase genes (reflected by different letter colouring) was based on phages infecting *L. curvatus* (dots and bold lettering) and *L. sakei*. The groups I to VI (including phages infecting *L. sakei*) were described in a previous study^[21]^. 1,000 replicates were used in the bootstrap analysis (Jukes-Cantor model). *L. curvatus*: *Latilactobacillus curvatus*.

We analysed the phylogenetic relationship between *L. curvatus* phage integrase genes in a similar fashion as previously described for *L. sakei* phage integrase genes^[21]^, which were included as outgroups in this analysis. This gave us additional information about the integration locus of each prophage. We adopted the previously found insertion groups I-VI and extended them by the *L. curvatus* phage-specific Groups VII-XIII [Figure 2]. *L. sakei* phages with group I integrases were all found integrated in tRNA genes for arginine and leucine. Group II phage integrases facilitated integration into tmRNA genes, group III into a gene with unknown function, group IV into a *sufB*-like gene, group V into a glutamine-hydrolysing GMP synthase gene, and group VI into a glucose-6-phosphate isomerase gene.

With the exception of group III integrases, all those groups obtained new *L. curvatus* phage members. Additionally, *L. curvatus* phages also integrated into a *lepA* gene (group VII; product: Translation elongation factor 4), and tRNA genes for serine (group VIII), glutamine (group IX), and glutamic acid (group X). Furthermore, two integrase clusters (group XI and Group XII) were found, which facilitate integration into non-coding regions.

Three outliers were found in which the integrase position in the phylogenetic tree did not reflect the chromosomal integration locus of the prophage. Phage TMW 1.2270 P1 contains a group II integrase, yet integrates into a tRNA gene for serine (normally group VIII). The other two outliers were phage members of group XIII (phage NFH-Km12 P1 and phage WiKim38 P2) integrating into a tRNA gene for leucine (normally group I). The integrases of group XIII share only 46.69% to 47.40% nucleotide similarity to the other integrases with leucine integration locus of group I, in contrast to 88.41% to 92.99% between group I integrases with leucine tRNAs as integration locus.

Notably, strain *L. curvatus* NFH-Km12 (here used as an example for both strains) has multiple genes coding for leucine tRNAs with percent identities ranging from 57.95% to 100% (100% when the same leucine tRNA gene is present in multiple copies). The leucine tRNA gene chosen for integration of phage NFH-Km12 P1 only shared a nucleotide similarity of 66.28% to 67.44% with group I leucine tRNAs in which the phages FLEC03 P1, TMW 1.1928 P1, and ZJUNIT8 P1 integrated (in contrast to 99.81% to 100.00% between the three leucine tRNA genes of group I). Therefore, the integrases of NFH-Km12 P1 and WiKim38 P2 were assigned to the new group XIII, despite also facilitating integration into leucine tRNA genes.

The determination of the chromosomal integration site of phage DRD-164 P1 was ambiguous. While the phage shared the att-sites with phages of group IX (insertion into tRNA-Gln gene), its respective attR-site was located before its lysis gene module. Another potential pair of att-sites was found (attL: TATTCGTTGATGATATT, attR: TATTCGTTGATCATTTT). It cannot be said with certainty if the phage is still intact.

A second gene (terminase large subunit) was analysed to further demonstrate the phylogenetic diversity between the intact prophages within this study [Supplementary Figure 3]. While no distinct clustering was observable when comparing the relationships between integrases with the relationship between terminase (large subunit) genes, the phylogenic relationship of terminase (large subunits) genes follows the relationship of prophages [Figure 1] more closely.

### Prophage inducibility

Screening for prophages in bacterial genomes provides insight into whether a species might be a target for temperate phages. However, phage functions such as the inducibility of the intact predicted prophages and correct virion assembly must be checked by performing induction experiments.

Six available lysogenic strains with intact predicted prophages were selected and exposed to one of two common phage inducers, UV light or mitomycin C. If induction and virion assembly were successful, host lysis and release of fully assembled virions would be expected. To assess the successful phage induction after the treatment, we monitored the growth of the lysogenic strains after induction treatment, analysed the lysates via transmission electron microscopy, and sequenced viral DNA from the potentially released virions.

**Figure 3:**
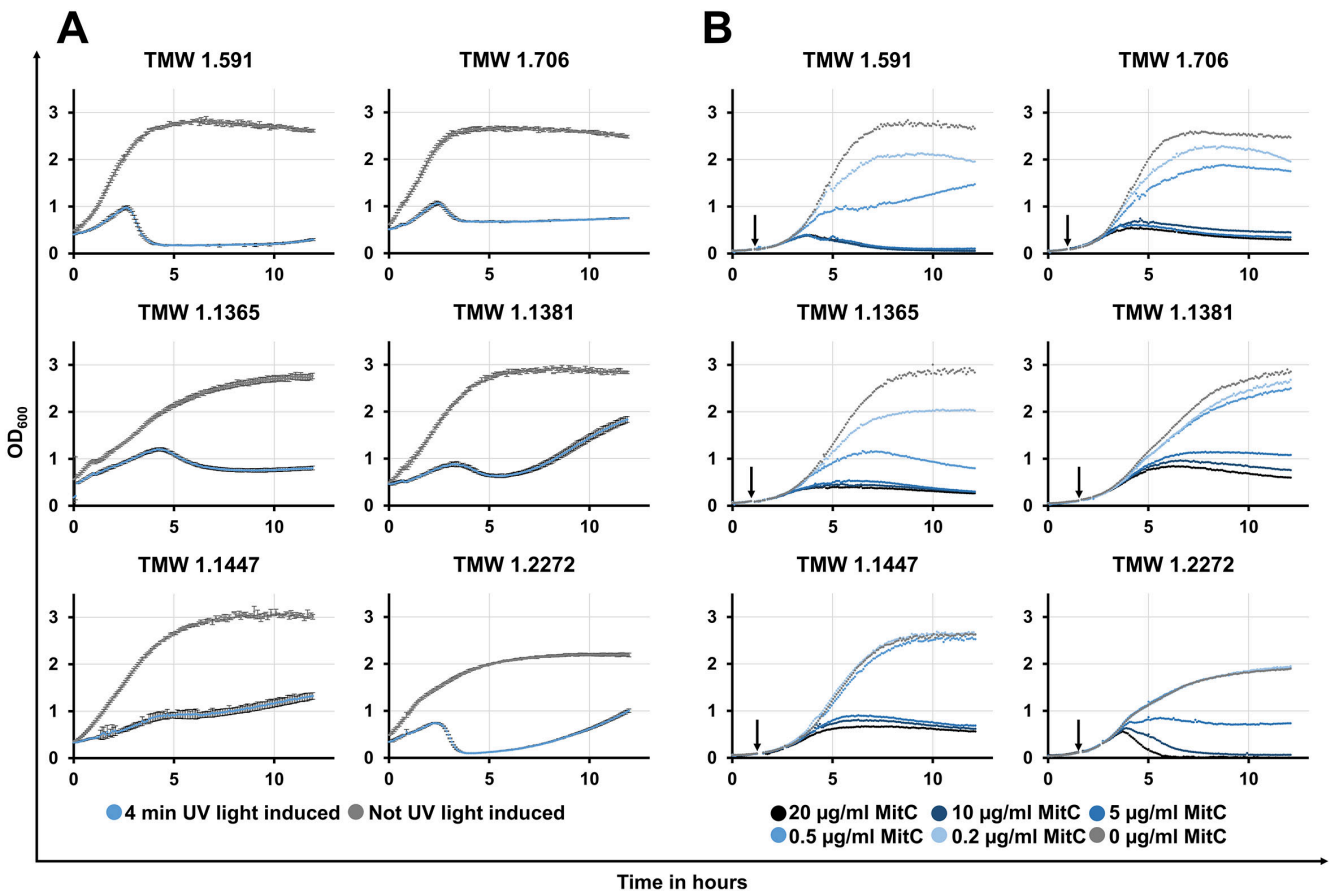
Growth curves of lysogenic *L. curvatus* strains after induction via UV light (A) or mitomycin C (B) treatment and without such treatment. Optical density was measured at 600 nm (OD_600_) (Y-axis; scale: 0-3.5) over the time (X-axis; scale 0-13) in hours (h). For the induction in (A), 4-min UV light exposure was used (no UV light exposure for control); For the induction in (B), different mitomycin C concentrations (20 µg/mL, 10 µg/mL, 5 µg/mL, 0.5 µg/mL, 0.2 µg/mL, and 0 µg/mL as control) were used. In (A), induction was performed directly before the measurement was started; In (B), the addition of mitomycin C to the bacterial cultures is indicated by an arrow. *L. curvatus*: *Latilactobacillus curvatus*.

As expected, all induced lysogens lysed after the induction process, although we experienced strain-dependent differences in lysis intensity and time [Figure 3]. Lysis started approximately 2-5 h after induction and ranged from a slight halt in growth (*L. curvatus* TMW 1.1447; UV light induction) to a rapid, nearly complete lysis of the bacterial culture (*L. curvatus* TMW 1.591; UV light induction).

**Figure 4:**
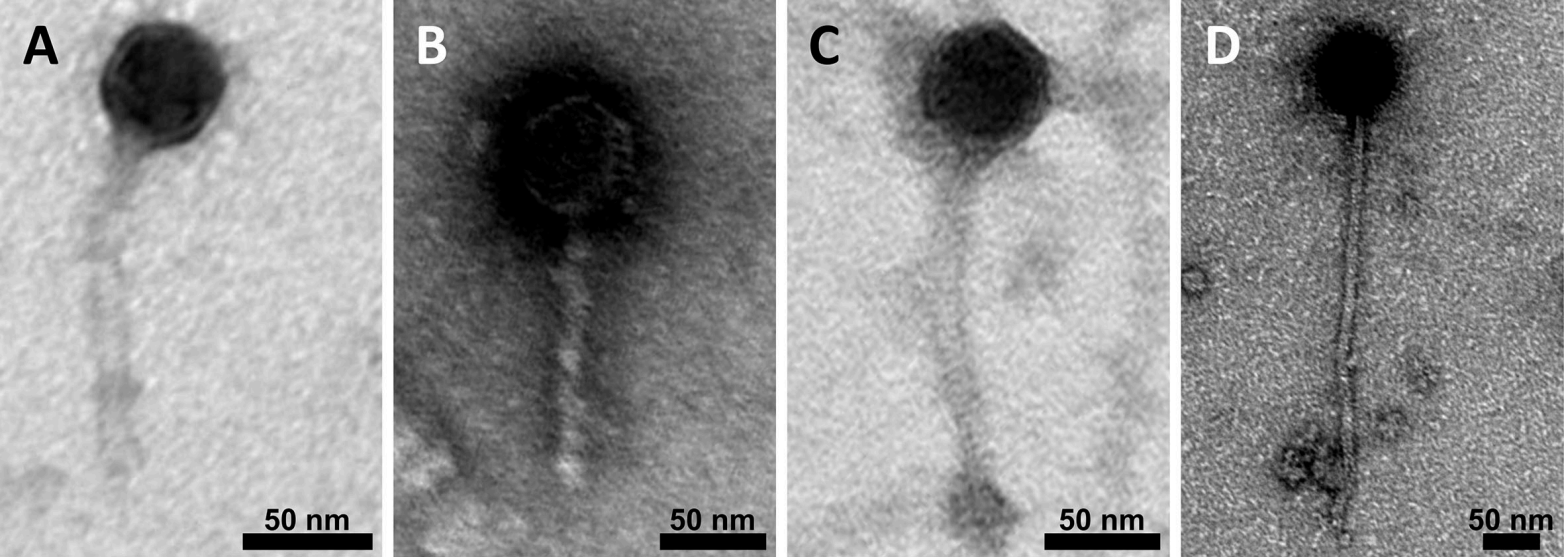
TEM derived micrographs of purified post-induction lysates of different *L. curvatus* strains. For negative staining of the samples, 2% uranyl acetate was used. (A) *L. curvatus* TMW 1.591; (B) *L. curvatus* TMW 1.706; (C) *L. curvatus* TMW 1.1365; (D) *L. curvatus* TMW 1.2272. Scale bar: 50 nm. The microscope was operated at 80 kV in zero-loss mode. TEM: Transmission electron microscopy. *L. curvatus*: *Latilactobacillus curvatus*.

The micrographs [Figure 4] showed virions with the siphovirus morphotype in four of the six lysate samples (A: Lysate of *L. curvatus* strain TMW 1.591, B: Lysate of *L. curvatus* strain TMW 1.706, C: Lysate of *L. curvatus* strain TMW 1.1365, and D: Lysate of *L. curvatus* strain TMW 1.2272). In the lysates of *L. curvatus* strains TMW 1.1381 and TMW 1.1447, no fully assembled virions were found. Notably, we could not identify different morphotypes of virions within one sample, even when multiple intact prophages were predicted in the same strain (e.g., *L. curvatus* TMW 1.706, and *L. curvatus* TMW 1.1365). While capsids (50-65 nm in diameter) and tail width (10-15 nm) were similar in size, tail lengths varied drastically between approximately 125-365 nm, with the shortest tail detected in the lysate of strain TMW 1.591 and the longest in the lysate of strain TMW 1.2272. Additional micrographs of purified post-induction lysates can be found in the supplemental data [Supplementary Figure 4].

As further proof of successful prophage induction, we extracted viral DNA from post-induction lysates after DNase I treatment. This ensures that host DNA is degraded, while viral DNA is protected within the phage capsids and is available for extraction and concentration. The viral DNA of all nine intact predicted phages was isolated from their corresponding lysate samples, although the quality of the sequenced genomes varied not only by strain but also by phage. Of the nine intact predicted phages, five were fully sequenced, three of them were marked as circular (phage TMW 1.1365 P2, phage TMW 1.1365 P3, and phage TMW 1.1381 P1), and two of them as linear (phage TMW 1.591 P1, phage TMW 1.706 P1). Alignments of isolated phage DNA with respective prophages are depicted in Supplementary Figure 5. Interestingly, the genome of phage TMW 1.1365 P2 contained a transposase that was previously not sequenced in its corresponding prophage. Three phage genomes were fragmented after assembly (phage TMW 1.706 P2, phage TMW 1.1.1365 P1, and phage TMW 1.2272 P1) with partially high depths of each phage-related fragment [phage TMW 1.706 P2 (depth = 5.26x to 8.02x), phage TMW 1.1.1365 P1 (depth = 235.19x to 330.19x), and phage TMW 1.2272 P1 (depth = 1238.18x to 2981.81x)]. Phage TMW 1.1447 P1 was sequenced as prophage but with a higher depth of its contig (depth = 131.29x). Fragmented phage genomes were completed by aligning to their corresponding prophage. A correlation between terminase genes and genome circularity, indicating potentially different packaging mechanisms, could not be unambiguously determined [Supplementary Figure 6].

## DISCUSSION

### Prophage incidence and integration

As a first step in understanding temperate phages infecting *L. curvatus*, we searched for prophages in openly available genomes (accessible on the NCBI web page) as well as genomes of strains from our in-house strain collection, where lysis was observable after an induction treatment with either UV light or mitomycin C.

A previous study by Pei *et al.* demonstrated that prophage distribution within the genus *Lactobacillus* is extremely uneven^[14]^. The authors suggested that multiple habitat species, such as *L. brevis*, *L. plantarum*, and *L. paracasei* would retain more intact prophages, both in terms of absolute count and incidence, in their genomes, than species occupying a smaller range of habitats. They identified *L. plantarum* as having the highest occurrence of intact prophages (98% of 134 analysed strains), and *L. paracasei* as the species with the highest “maximum number of prophage fragments” (15) and highest mean in “number of prophage fragments” (4.39 per strain; analysed strains: 147)^[14]^.

Comparing this data to the results of our study, we found that *L. curvatus* can also reach high numbers (up to 14) in prophage count (discounting completeness) and has a mean of 4.6 prophages per strain (206 prophage sequences in total over 45 genomes analysed). This would therefore be an even higher value than what can be found for *L. paracasei* but with a lower count of “maximum intact prophages” (three for *L. curvatus* in contrast to five for *L. paracasei*) at a similar intact prophage occurrence (73.3% of analysed *L. curvatus* strains harbouring intact prophages in contrast to 72.1% in *L. paracasei*)^[14]^. Notably, the six strains included for the prophage induction experiments were also included in the predictive analyses, which may have increased the overall number of intact predicted prophages to some extent.

*L. curvatus* seems to harbour more prophages than *L. sakei* (maximum prophage sequence count: 5; Mean: 2.43; Occurrence intact prophages: 65.9%)^[14]^, a species known for sharing a phylogenetically and phenotypically close relationship with *L. curvatus* to such an extent that rapid differentiation between the two species was historically challenging^[44]^. According to Pei *et al.*^[14]^, this might be explained by a less restricted habitat *L. curvatus* occupies in contrast to *L. sakei*, even though both species share some ecological niches, such as meat and fermented meat products^[16-20]^.

In total, of the 13 different distinct chromosomal loci we identified for *Latilactobacillus* phages (*L. sakei* and *L. curvatus*), intact *L. curvatus* prophages were integrated into twelve of them, mostly in genes known for their conservative nature (i.e., tRNA genes^[45]^, *lepA* coding for translation elongation factor 4^[46]^). Conserved genes are often targeted by phages as integration loci, and our results are therefore consistent with the literature^[47]^. It should be noted that, in some cases, an exact prediction of the att-sites was hindered by ambiguous BLAST results or incomplete sequencing data.

### Genomic composition and features of *L. curvatus* prophages

The intact prophages we found in *L. curvatus* are genomically similarly organized compared to *L. sakei* prophages and share similar integrase genes, and thus also chromosomal integration loci^[21]^. Next to *L. sakei* prophages, the correlation between closely related integrase genes and identical chromosomal integration loci has also been demonstrated for *Levilactobacillus brevis* temperate phages^[7]^. When comparing closely-related phages, highly conserved genetic regions with > 63% nucleotide similarity were mainly found in replication and head/tail-related genes. Putatively identical prophages were only harboured by *L. curvatus* strain TMW 1.706 and the type strain *L. curvatus* DSM 20019^T^. This can be explained by the overall high percentage identity of the genomes of both strains (100.00% ANIb over 99.95% of aligned nucleotides). The average nucleotide consistency of all intact predicted prophages is depicted as a pairwise comparison table in the supplemental material [Supplementary Figure 1].

Some of the found prophages harboured features that were not ubiquitously present in all of them. Of course, the partial presence or lack of some of these features in those prophages might be explained by poor annotation/database entries. Here, we discuss the presence and putative function of transposases, methyltransferases, potential modification systems (methyltransferases in combination with restriction endonucleases), and tRNAs.

Transposases were located in five of the intact predicted prophages transposases. Whether these phage genomes are truly intact is ambiguous; on one hand, these phage genomes still have the potential to be intact; however, on the other hand, transposases can replace important genes for further propagation, as shown in *Neisseria meningitidis*, where an IS30 transposase replaced the prophage-related head and tail morphogenesis genes^[48]^. Bobay *et al.* discussed the rapid deletion of prophage genes, not only as a fast protective mechanism by the host but also as an early step to “domesticate” phage components for the host’s usage^[49]^. In the genome of phage TMW 1.1365 P2, a putative transposase was located after the sequencing of the post-induction lysate, which was not present in the prophage. We therefore suggest that transposases can indeed be tolerated to some extent during phage induction, with the prerequisite that no essential genes are disrupted/lost, although they might also be an indication for the ongoing degradation of the prophage.

34% of intact predicted prophages contained tRNA genes (excluding partial tRNA genes used for integration). The presence of tRNA genes in phage genomes was discussed to support translation during the degradation of the host chromosome after infection with lytic vibriophage 2.275.O^[50]^. Furthermore, tRNA genes provided by the phage are discussed to potentially compensate for codon usage of the host^[51]^. Yoshikawa *et al.* demonstrated for Xanthomonas citri jumbo phage XacN1, that codon usage varies between phage XacN1 and its host and that the phage-encoded tRNAs target codons used at a higher frequency by the phage than its host^[52]^.

The intact predicted *L. curvatus* (pro)phages of this study harboured predominantly isoleucine tRNA genes, in which 7/10 use the CAU anticodon. Tomikawa *et al.* have shown that, for *Lactiplantibacillus plantarum*, the isoleucine tRNA using the CAU anticodon [tRNA^Ile2^(CAU)] is post-transcriptionally modified by the enzyme tRNA^Ile^-lysidine synthetase (TilS) to produce tRNA^Ile2^(LAU), which decodes the minor AUA isoleucine codon^[53]^. Therefore, the high frequency of tRNA genes using the CAU anticodon in *L. curvatus* prophages could be explained by the higher frequency of the minor AUA isoleucine codon used in the coding regions of those phages in contrast to the coding regions of their hosts. Furthermore, all *L. curvatus* strains with isoleucine tRNA gene harbouring intact prophages also carry the aforementioned *tilS* gene. The hypothesis of *L. curvatus* phages bringing tRNA genes to offset the codon usage of their host is tentatively supported by these results for this specific isoleucine tRNA. In contrast, *L. curvatus* (pro)phages harbouring phenylalanine tRNA genes that exclusively use GAA as anticodon used the corresponding codons at a similar rate as their hosts. It should be noted that the number of samples analysed in these comparisons was relatively small [Phage/host genomes with respective tRNA-harbouring phages: n(tRNA^Ile^) = 8; n(tRNA^Phe^) = 7] due to the limited number of accessible genomes for *L. curvatus*. As more genomes become available for this species, more robust results can be obtained.

If the differences in codon usage between phage and host increase the amount of corresponding tRNA genes in phage genomes, this phenomenon could theoretically also depend on the number of corresponding tRNA genes within the host genome and the particular tRNA gene itself. In a previous study in *Escherichia coli*, McDonald *et al.* analysed the retention of tRNA genes as a consequence of rapid influxes of maladapted, foreign genes through lateral gene transfer, for example, via temperate phage infection/prophage integration^[54]^. The authors concluded that tRNA genes might selectively be kept by the host to accommodate shifting demands in codon usage^[54]^. Therefore, the transduction of tRNA genes by intact predicted prophages in our study could partially support the observed differences in codon usage, such as for the isoleucine codon AUA, although corresponding tRNA genes could also originate from other sources.

Interestingly, methyltransferase (MTases) genes were also annotated in some of the intact predicted *L. curvatus* prophages. These MTases generally can occur alone, so-called “orphan” MTases, or in combination with restriction endonucleases (REases), forming a restriction-modification system (R-M system)^[55]^. While R-M systems can be found in most bacterial and archaeal genomes as a protection mechanism against foreign DNA (e.g., viral genomes, or transposons)^[56]^, MTases are sometimes used by phages to protect their genomes against R-M systems after host infection^[57]^. In our analyses, we mainly found “orphan” MTases within the prophages of *L. curvatus*, although three prophages (phage FAM25164 P1, phage MRS6 P1, and phage TMW 1.591 P1) harboured genes annotated as “integrase (tyrosine recombinase)”, or “HNH homing endonuclease” directly downstream of those MTases.

For Escherichia coli phage P1, DNA methylation has been demonstrated to have a positive effect on the efficiency of DNA packaging into the phage capsid^[58]^. The question arises whether the supportive function of DNA methylation is a general requirement for phage DNA packaging during the lytic cycle, and whether this also applies to phages infecting *L. curvatus*. If this were the case, MTases should be expected in all intact predicted prophages in this study. Three potential explanations could address this observation, which requires future investigation. DNA methylation might (i) not be a ubiquitous requirement during phage packaging; (ii) methylation might be performed by MTases provided by the host; and (iii) MTases are present, but annotation failed due to poor matching with databases. To verify any of these options, methylome data would be required, which is still an expensive venture, although high-throughput methods are on the rise^[59]^. From a host perspective, the presence of MTases is also very interesting, as methylation has been shown to play an important role in the DNA repair of *E. coli*^[60,61]^. A previous study about Burkholderia mallei phage ΦE125, whose genome encodes two DNA MTase genes, demonstrates the specific methylation of its phage episome, neither host chromosome nor prophage. The authors conclude that methylation of extrachromosomal phages must play an important role in their life cycle and is likely connected to their replication rate^[62]^. It is still unknown whether the methyltransferases of phages in this study can interact with the host chromosome, regardless of prior induction.

We could not identify antibiotic resistance genes (ARG) transduced by the intact predicted prophages. Wendling *et al.* have shown that prophages can benefit the fitness of their host independent of the presence of phage-provided ARGs^[9]^. The authors hypothesised that this may be caused by the release of virions capable of lysing susceptible competitors, and that the competitiveness of lysogens, in turn, could depend on and vary with the inducibility of prophages in their specific environment^[9]^. This inducibility was proven for some of the *L. curvatus* prophages in this study.

### Prophage inducibility

We supported our predictive approaches with induction experiments, which demonstrated the manifold lysis responses of different putative lysogens ranging from only a small step in the post-induction growth curve (*L. curvatus* strain TMW 1.1447) to nearly complete lysis (*L. curvatus* strain TMW 1.2272). We used the two common phage inducers, UV light and mitomycin C, to induce prophages within six *L. curvatus* lysogens. Both inducers cause DNA damage to the host, activating its SOS response system and resulting in conversion from lysogenic to lytic life cycle of phages, as shown in *E. coli*^[63]^.

Notably, we wanted to show that induction is possible with both induction treatments, as each method has its advantages. Mitomycin C might be the easier method to transfer and reproduce induction between laboratories, yet UV light can be a powerful, cheap, and accessible alternative to antibiotics such as mitomycin C. We achieved the best results using a four-min UV light treatment for induction; this protocol was also used for the post-induction growth experiment depicted in Figure 3 and previously for phage induction in *L. sakei*^[21]^. When applied for the first time, various UV irradiation durations and/or intensities should be tested. We found that UV light was a preferable induction method over mitomycin C. Following induction treatment, the growth curves of all tested lysogenic strains displayed a halt or decrease in turbidity measured at an optical density at 600 nm (OD_600_), indicating lysis and suggesting successful prophage induction. Lysis differences also occurred between different strains with only one intact prophage (e.g., *L. curvatus* TMW 1.591 and *L. curvatus* TMW 1.1447). We conclude that the lysis response is strongly phage/host-dependent and not necessarily reliant on the number of intact prophages in a specific strain.

Only virions with the siphovirus morphotype (icosahedral capsids and long non-contractile tails) were found in the micrographs of the purified, post-induction lysates. Notably, only one phage morphotype was found per lysate of each induced lysogenic *L. curvatus* strain, although multiple intact prophages were predicted for some of them (*L. curvatus* strains TMW 1.706 and TMW 1.1365), similar to our previous study describing prophages in *L. sakei*^[21]^. There are multiple possible explanations for this. It is possible that (i) only some of the intact predicted prophages have been extensively induced; or (ii) induction happened for all prophages, but only one phage particle morphotype was assembled. The sequencing of viral DNA extracted from post-induction analyses verified that multiple prophages were indeed induced simultaneously, although at least one phage genome of multi-prophage harbouring strains was fragmentized after genome assembly. This could potentially indicate different amounts of viral DNA substrate for sequencing. Contrary to this argument, *L. curvatus* phage TMW 1.2272 P1, which caused a strong lysis event after induction and appeared to generate fully assembled virions as detected via TEM, had a fragmentized viral genome after assembly as well, despite being the only prophage within *L. curvatus* strain TMW 1.2272. The exact reasons for genome fragmentation and the presence of only one phage morphotype per lysate are still unknown and may need further attention.

## CONCLUSION

Our analyses for the commonly used starter organism *L. curvatus* affirmed the close relationship between temperate phages and lactobacilli and revealed 50 putatively intact prophages located in 12 discrete chromosomal loci. Knowledge of conserved regions within phage genomes and integration loci could be useful to design a prophage detection method (e.g., a PCR-based approach) that is not reliant on induction experiments. Some of the intact predicted phages contain features, such as genes coding for transposases, MTases, and tRNAs, which demonstrate their diversity as well as indicate their battle against their host. We supported our predictive analyses by demonstrating that, after induction treatment with UV light or mitomycin C, some of these viral sequences can influence the growth of their hosts in varying intensities, accompanied by the release of new phage progeny with the siphovirus morphotype. Therefore, we suggest that these lysogens might also have the potential to influence other microbial communities.

## DECLARATIONS

### Availability of data and materials

Accession numbers of *L. curvatus* genomes used in this study are listed below. The outgroups used genomic data are listed with the respective accession numbers, and/or locus tags in the supplemental material.

*L. curvatus* genomes (accessible at the NCBI website) used for prophage screening analyses:

CBA3617 (NZ_CP042389), CRL 705 (NZ_AGBU01000145), DRD-164 (JAMRWB000000000), ELA204092 (NZ_JAJJOT010000000), ELA214002 (NZ_JAJJOL010000000), ELA214060 (NZ_JAJJOQ010000000), ELA214061 (NZ_JAJJOR010000000), ELA214062 (NZ_JAJJOS010000000), ELA214117 (NZ_JAJJOK010000000), ELA214388 (NZ_JAIULU010000000), FAM25164 (NZ_JAFJMA010000001), FBA2 (NZ_CP016028), IRG2 (NZ_CP025476), isolate FLEC03 chromosome LCUFL03 (NZ_LT841333), HFS9 (NZ_JAMOHH010000001), JCM 1096 = DSM 20019^T^ (NZ_CP026116), KG6 (NZ_CP022475), MGYG-HGUT-00020 (NZ_CABIVZ010000001), MRS6 (NZ_CP022474), NFH-Km12 (AP018699), NRIC0822 (NZ_JTJV01000001), RI-124 (NZ_MKDR01000001), RI-406 (NZ_MKDG01000001), S46 (NZ_SUMW01000010), SRCM103465 (NZ_CP035110), TMW 1.27 (CP016467), TMW 1.401 (CP016216), TMW 1.407 (CP016218), TMW 1.421 (CP016221), TMW 1.595 (CP016470), TMW 1.624 (CP015490), TMW 1.1381 (CP015493), TMW 1.1390 (CP015494), TMW 1.1408 (JAHIAF000000000), TMW 1.1928 (NZ_CP031003), TMW 1.2270 (JAHIAD000000000), VRA_2sq_f (NZ_WKLA01000324), WDN19 (NZ_AP024685), WiKim38 (NZ_CP017124), ZJUNIT8 (NZ_CP029966).

*L. curvatus* genomes provided by this study:

TMW 1.591 (JAHIAT000000000), TMW 1.706 (JAHIAS000000000), TMW 1.1365 (JAHIAR000000000), TMW 1.1447 (JAHIAQ000000000), TMW 1.2272 (JAHIAP000000000).

Viral genomes provided by this study:

Phage_TMW_1.591_P1 (OQ240252), Phage_TMW_1.706_P1 (OQ240253), Phage_TMW_1.706_P2 (OQ240254), Phage_TMW_1.1365_P1 (OQ240255), Phage_TMW_1.1365_P2 (OQ240256), Phage_TMW_1.1365_P3 (OQ240257), Phage_TMW_1.1381_P1 (OQ240258), Phage_TMW_1.1447_P1 (OQ240259), Phage_TMW_1.2272_P1 (OQ240260).

### Copyright

The Author(s) 2023.
