# Supplementary material for: Distribution, inducibility, and characteristics of Latilactobacillus curvatus temperate phages
Source: Microbiome Res Rep. 2023 Sep 8;2(4):34. doi: 10.20517/mrr.2023.18 (PMC10688831; doi:10.20517/mrr.2023.18)
Supplement: Supplementary file 1 [file mrr-2-4-34-SupplementaryMaterials.pdf]

Supplemental Material: "Distribution, Inducibility, and Characteristics of *Latilactobacillus curvatus* Temperate Phages"

Supplementary Table 1. Excluded *L. curvatus* strains due to high genome similarity (ANIb values [%] and aligned nucleotides (AP) [%] listed from perspective of excluded strain).

L. curvatus strain included	*L. curvatus* strain excluded	ANIb [%]	AP [%]
DRD-164 (JAMRWB000000000)	DRD-170 (JAMRWA010000000)	100.00	98.55
	DRD-171 (JAMRVZ010000000)	100.00	98.67
ELA204092 (NZ_JAJJOT010000000)	ELA204023 (NZ_JAJJOM010000000)	100.00	99.85
	ELA204033 (NZ_JAJJOO010000000)	99.98	99.57
	ELA204093 (NZ_JAIULW010000000)	99.99	95.77
	ELA204096 (NZ_JAJJOU010000000)	100.00	99.79
ELA214002 (NZ_JAJJOL010000000)	ELA204029 (NZ_JAJJON010000000)	99.99	99.63
	ELA204098 (NZ_JAJJOV010000000)	100.00	99.69
	ELA204100 (NZ_JAIULV010000000)	100.00	96.47
ELA214060 (NZ_JAJJOQ010000000)	ELA214059 (NZ_JAJJOP010000000)	99.98	99.87
TMW 1.706 (JAHIAS000000000)	NBRC 15884 (NZ_BJOQ01000001)	99.98	99.30
TMW 1.1381 (CP015493)	RI-193 (NZ_MKGD01000001)	99.99	99.73
	RI-198 (NZ_MKGC01000001)	99.99	99.67
	TMW 1.167 (CP016472)	99.98	99.77
	TMW 1.439 (CP015489)	99.98	99.91
VRA_2sq_f (NZ_WKLA01000324)	VRA_2sq_n (NZ_WKKT01000028)	99.99	99.58

Supplementary Table 2. *L. curvatus* strains analysed in this study (for accession numbers see chapter "Availability of data and materials), the number of prophage sequences in strains, where genome data was available, including completeness evaluation of those prophages (i, intact; q, questionable; inc, incomplete) as predicted by PHASTER.

***L. curvatus* strain**	**Isolation source (origin)**	**Number of detected prophages**		
		**i**	**q**	**inc**
CBA3617	Kimchi (South Korea)	3	1	6
CRL 705	Argentinian dry fermented sausage (Argentina)	0	0	2
DRD-164	Artisanal Greek feta cheese (Greece)	3	1	0
ELA204092	Atlantic salmon (Norway)	0	0	2
ELA214002	Atlantic salmon (USA)	0	0	1
ELA214060	Atlantic salmon (USA)	0	0	1
ELA214061	Atlantic salmon (USA)	0	1	1
ELA214062	Atlantic salmon (USA)	0	0	2
ELA214117	Atlantic salmon (USA)	2	0	1
ELA214388	Atlantic salmon (USA)	2	0	11
FAM25164	Cheese (Switzerland)	1	0	1
FBA2	Radish and carrot pickled with rice bran and salt (Japan)	0	0	6
FLEC03 LCUFL03	Beef carpaccio (-)	1	0	1
HFS9	Human faeces (China)	0	1	1
IRG2	Human faeces (South Korea)	1	4	1
JCM 1096 = DSM 20019^T	Milk (Germany) – Type strain	3	0	4
KG6	Salami (Switzerland)	1	3	2
MGYG-HGUT-00020	Human gut (-)	0	0	2
MRS6	Fermented sausage salsiz (Switzerland)	1	0	0
NFH-Km12	Kabura-zushi (Japan)	0	1	8
NRIC0822	Kabura-zushi (Japan)	1	0	2
RI-124	Food (-)	0	0	1
RI-406	Food (-)	1	0	0
S46	Cattle (Canada)	0	0	2
SRCM103465	Food (South Korea)	1	0	1
TMW 1.27	-	0	1	2
TMW 1.401	Sauerkraut (Germany)	0	1	4

TMW 1.407	Sauerkraut (Germany)	1	2	3
TMW 1.421	-	1	1	10
TMW 1.591	-	1	0	0
TMW 1.595	-	1	2	4
TMW 1.624	Italian raw sausage (Germany)	3	4	1
TMW 1.706	Sourdough (Germany)	2	0	4
TMW 1.1365	-	2	0	4
TMW 1.1381	Salami starter culture (Germany)	0	6	3
TMW 1.1390	Salami starter culture (Germany)	2	1	1
TMW 1.1408	Fermented fresh water fish (Norway)	1	0	1
TMW 1.1447	Pecorino cheese (Sardinia)	1	0	0
TMW 1.1928	Italian raw sausage (Italy)	2	2	10
TMW 1.2270	Rye sourdough (Germany)	1	1	2
TMW 1.2272	Mealworm (Germany)	1	0	1
VRA_2sq_f	Pheasant (Russia)	3	0	0
WDN19	Japanese pickle (Japan)	2	3	5
WiKim38	Baechu-Kimchi (South Korea)	2	1	5
ZJUNIT8	Chinese pickle (China)	0	1	9

Supplementary Table 3. Integration sites of intact predicted *L. curvatus* phages. Listed are the attachment sites attL and attR of each phage, as well as the gene products and the locus tags of the genes where those phages integrate.

Phage	attL (integrase site)	attR (lysin site)	Integration site or gene product	Locus tag
**CBA3617 P1**	AATATTTTTAAAA	TTTTAAAAATATT*	non-coding region	non-coding region
**CBA3617 P2**	ATTGGGTATATAGG	ATTGGGTATATAGG	tRNA-Gln	FGL79_RS09100
**DRD-164 P1**	ATTGGGTATATAGG<br><br>alternatively: TATTCGTTGATGATATT	ATTGGGTATATAGG<br><br>alternatively: TATTCGTTGATCATTTT	tRNA-Gln<br><br>alternatively: FtsX-like permease family protein	NE283_RS06015<br><br>alternatively: NE283_RS06260
**DRD-164 P2**	AGTAAGGAGAGTACAGGATT	AGTAAGGAGAGTACAGGATT	tRNA-Ser	NE283_RS08625
**DSM 20019 P1**	AAAAGTTACCACATAAATTACCACA	AAAACTTACCACATAATTTACCACA	non-coding	non-coding
**DSM 20019 P2**	ATTGGGTATATAGG	ATTGGGTATATAGG	tRNA-Gln	LCU_RS03995
**ELA214061 P1**	AGTAAGGAGAGTACAGG	AGTAAGGAGAGTACAGG	tRNA-Ser	LOX59_RS00460
**ELA214117 P1**	AGGAGAGTACAGG	AGTAAGGAGAGTACAGG	tRNA-Ser	LOX61_RS00435
**ELA214117 P2**	TGTTTTAAATATCACCCGTACGG	TGTTTTAAATATCACCCGTACGG	tRNA-Glu	LOX61_RS01630
**ELA214388 P1**	TATGAAAAAATG	CATTTTTTCATA*	*sufB*<br>product: Fe-S cluster assembly protein SufB<br><br>and bacteriophage abortive infection AbiH family protein	LBW12_RS02485<br><br>LBW12_RS02730
**ELA214388 P2**	CCCGGGTGACGCATATATA	CCCGGGTGACGCATATATA	tRNA-Arg	LBW12_RS05205
**FAM25164 P1**	CCTGTACTCTCCTTACT	CCTGTACTCTCCTTACT	tRNA-Ser	JYG89_RS00990
**FLEC03 P1**	ATGCCCGTGGCAGG	ATGCCCGTGGCAGG	tRNA-Leu	LCUFL03_RS07095
**HFS9 P1**	TAAGGAGAGTACAGG	TAAGGAGAGTACAGG	tRNA-Ser	NB814_RS01100
**IRG2 P1**	CCTGTACTCTCCTTACT	CCTGTACTCTCCTTACT	tRNA-Ser	CYK59_RS03750
**KG6 P1**	ATTGGGTATATAGG	ATTGGGTATATAGG	tRNA-Gln	CGZ47_RS06095
**MRS6 P1**	AGAAGCCTTCATGTCCATT	AGAAGCCTTCATGTCCATT	*lepA*	CG419_RS03715

			product: translation elongation factor 4	
**NFH-Km12 P1**	ATTATACCGGTGATCGG	ATTATACCGGTGATCGG	tRNA-Leu	NFHkm12_t00360
**NRIC0822 P1 joined sequences**	AACAGTGGAATGGGAATAGA	n.d.**	*guaA*<br>product: glutamine-hydrolyzing GMP synthase	OA78_RS02880
**RI-406 P1**	ATTCTATTCCCATTCCACTGTT	ATTTTATTCCCATTCAATTGTT	*guaA*<br>product: glutamine-hydrolyzing GMP synthase	B0D99_RS01070
**SRCM103465 P1**	AGAAGCCTTCATGTCCATT	AGAAGCCTTCATGTCCATT	*lepA*<br>product: translation elongation factor 4	EQK21_RS03855
**TMW 1.27 P1**	AAACGTTGAATTATA	AAACGTTGAATTATA	*pgi*<br>product: glucose-6-phosphate isomerase	A4W75_04750
**TMW 1.27 P2**	AATGGAGCCGGCG	AATGGAGCCGGCG	*ssrA* (tmRNA-gene)	A4W75_05470
**TMW 1.591 P1**	ATTGGGTATATAGG	ATTGGGTATATAGG	tRNA-Gln	KNP41_00865
**TMW 1.595 P1**	CCTATATACCCAAT	CCTATATACCCAAT	tRNA-Gln	A4W80_04695
**TMW 1.624 P1**	CGCCTGTGACATTCAA	CGCCTGTGACATTCAA	tRNA-Arg	A4W72_02985
**TMW 1.624 P2**	ATTGGGTATATAGG	ATTGGGTATATAGG	tRNA-Gln	A4W72_06160
**TMW 1.706 P1 joined sequences**	ATTGGGTATATAGG	ATTGGGTATATAGG	tRNA-Gln	KNP59_03170
**TMW 1.706 P2 joined sequences**	AAAAGTTACCACATAAATTACCACA	AAAACTTACCACATAATTTACCACA	non-coding	non-coding
**TMW 1.1365 P1**	ATGCGTCACCCGGGAGT	ATGCGTCACCCGGGAGT	tRNA-Arg	KNP66_00315
**TMW 1.1365 P2**	TATCCTACAGACCCTTCCA	n.d.	*sufB*<br>product: Fe-S cluster assembly protein SufB	KNP66_03450
**TMW 1.1365 P3**	TTATTTACCGGCTTTATCTTCATCATTCATTTTAAGAATGGACATGAACGCCTCT	TTATTTACCTTTGGTATCTTCATCATTCATCTTCAAAATGGACATGAACGCCTCT	*lepA*<br>product: translation elongation factor 4	KNP66_05290
**TMW 1.1381 P1**	TTGAATGTCACAGGCG	TTGAATGTCACAGGCG	tRNA-Arg	A4W73_07170
**TMW 1.1390 P1**	AGTAAGGAGAGTACAGG	AGTAAGGAGAGTACAGG	tRNA-Ser	A4W74_06765
**TMW 1.1390 P2**	CGCCTGTGACATT	CGCCTGTGACATT	tRNA-Arg	A4W74_07405
**TMW 1.1408 P1**	ATGCGTCACCCGGGAGT	ATGCGTCACCCGGGAGT	tRNA-Arg	KNO77_02635
**TMW 1.1447 P1**	n.d.	n.d.	n.d.	n.d.
**TMW 1.1928 P1**	CCTGCCACGGGCAT	CCTGCCACGGGCAT	tRNA-Leu	DT351_RS03180
**TMW 1.1928 P2**	AATGTCACAGGCG	AATGTCACAGGCG	tRNA-Arg	DT351_RS07515
**TMW 1.2270 P1**	AATCCTGTACTCTCCTT	AAGCCTGTACTCTCCTT	tRNA-Ser	KNO48_01595
**TMW 1.2272 P1**	AACAGTGGAGTGGGAATA	AACAATTGAGTGGGAATA	*guaA*<br>product: glutamine-hydrolyzing GMP synthase	KNP65_06740
**VRA_2sq_f P1 = P3**	TAAGGAGAGTACAGG	TAAGGAGAGTACAGG	n.d.<br>(potentially in serine tRNA gene)	n.d.
**VRA_2sq_f P2**	ATTAAACGAACGCCTATAA	ACTAAACGAACGCCTATAA	glucose-6-phosphate isomerase	GKC32_RS00475
**WDN19 P1**	CCTATATACCCAAT	CCTATATACCCAAT	tRNA-Gln	LTWDN19_RS00245

**WDN19 P2**	AATGGTCGTACATAA ATATTTTTAAAA	TTTTAAAAATATTAG TGAACTGCCATT	non-coding	non-coding
**WDN19 P3**	TACAAGAAGAACATG TTTGC	TACAAGAAGAACATG TTTGC	glucose-6-phosphate isomerase	LTWDN19_RS1 0095
**WiKim38 P1**	AATATTTTTAAAA	TTTTAAAAATATT	non-coding	non-coding
**WiKim38 P2**	ATTATACCGGTGATC GG	ATTATACCGGTGATC GG	tRNA-Leu	LCW_RS07950
**ZJUNIT8 P1**	CCTGCCACGGGCAT	CCTGCCACGGGCAT	tRNA-Leu	C0W45_RS0282 5
**ZJUNIT8 P2**	AATGTCACAGGCG	AATGTCACAGGCG	tRNA-Arg	C0W45_RS0696 5

*attR sequence is the reverse-complement to attL sequence

**n.d.: Not determinable due to missing sequence information

Supplementary Table 4. General features of *L. curvatus* prophages (length, number of coding regions (CDS), GC-content, number of tRNA genes, and genomic locus).

Phage	Length [bp]	CDS	GC-content [%]	tRNA genes (additionally used for integration)	genomic locus (accession number/contig, bases from...to)
CBA3617 P1	35282	55	39,9	0	NZ_CP042389, complement(join(1896984..1915352,1..16913))
CBA3617 P2-RC[*1]	39147	56	39,6	2 (1)	NZ_CP042389, complement(1731973..1771119)
DRD-164 P1	34087	47	39,5	1 (1)	NZ_JAMRWB010000001, 1167675..1201761
DRD-164 P2-RC	29059	49	40,2	0 (1)	NZ_JAMRWB010000001, complement(1628643..1657701)
DSM 20019 P1	35594	53	40,2	1	NZ_CP026116, 424333..459926
DSM 20019 P2-RC	40878	55	39,2	0 (1)	NZ_CP026116, complement(702468..743345)
ELA214061 P1-RC	40338	59	40,1	2 (1)	NZ_JAJJOR010000001, complement(26454..66791)
ELA214117 P1-RC	39949	55	39,6	1 (1)	NZ_JAJJOK010000001, complement(26487..66435)
ELA214117 P2	38274	62	41,9	0 (1)	NZ_JAJJOK010000002, 52155..90428
ELA214388 P1	36561	49	40,6	0	NZ_JAIULU010000001, 485049..521609
ELA214388 P2-RC	37678	53	38,9	0 (1)	NZ_JAIULU010000001, complement(1023191..1060868)
FAM25164 P1	37926	56	40,4	0 (1)	NZ_JAFJMA010000002, 84044..121969
FLEC03 P1-RC	37192	49	39,7	1 (1)	NZ_LT841333, complement(1344331..1381522)
HFS9 P1-RC	39854	57	40,6	0 (1)	NZ_JAMOHH010000002, complement(602..40455)
IRG2 P1	42008	58	39,8	1 (1)	NZ_CP025476, 693056..735063
KG6 P1-RC	29420	40	39,4	1 (1)	NZ_CP022475, complement(1152128..1181547)
MRS6 P1	51237	71	38,3	2	NZ_CP022474, 740806..792042
NFH-Km12 P1-RC	36081	53	38,2	0 (1)	AP018699, complement(1508477..1544557)
NRIC0822 P1 joined sequences[*2]	43843	58	39,3	1	P1.1: NZ_JTJV01000015, 33699..47934; P1.2: NZ_JTJV01000021, 1..29607
RI-406 P1-RC	44647	68	39,3	1	NZ_MKDG01000003, complement(5063..49709)

SRCM103465 P1	40624	61	40,0	1	NZ_CP035110, 763448..804071
TMW 1.27 P1	44831	63	39.2	0	CP016467, complement(905943..950773)
TMW 1.27 P2	35600	47	41.1	0	CP016467, complement(1053370..1088969)
TMW 1.591 P1	38227	52	41,0	0	1, complement(115313..153539)
TMW 1.595 P1	34120	40	39.8	0	CP016470, 913930..948049
TMW 1.624 P1	36943	59	42.3	0 (1)	CP015490, 578300..615242
TMW 1.624 P2	36384	49	39.8	0 (1)	CP015490, complement(1134239..1170622)
TMW 1.706 P1 joined sequences	39909	55	39,1	0	P1.1: 8, complement(146..17188); P1.2: 11, complement (42792..65657)
TMW 1.706 P2 joined sequences	34756	52	40,2	0	P2.1: 15, 45596..57594; P2.2: 16, complement (32242..55012)
TMW 1.1365 P1	30500	51	43,5	0 (1)	1, 11430..41929
TMW 1.1365 P2	35394	54	40,4	0	5, complement(1..35394)
TMW 1.1365 P3	45756	71	39,1	0	9, complement(5148..50903)
TMW 1.1381 P1	32337	55	42,2	0 (1)	CP015493, complement(1350810..1383146)
TMW 1.1390 P1	36714	54	40.5	2 (1)	CP015494, complement(1276640..1313353)
TMW 1.1390 P2	36833	51	39.6	0 (1)	CP015494, complement(1385297..1422129)
TMW 1.1408 P1	33598	55	42,0	0 (1)	4, 7850..41447
TMW 1.1447 P1	38073	58	40,0	0	15, 1..38073
TMW 1.1928 P1	36244	48	39,3	0 (1)	NZ_CP031003, 605975..642218
TMW 1.1928 P2-RC	33138	55	41,6	0 (1)	NZ_CP031003, complement(1401111..1434248)
TMW 1.2270 P1	38163	51	38,7	0 (1)	2, 109923..148085
TMW 1.2272 P1	41125	53	40,3	0	9, 36126..77250
VRA_2sq_f P1 = P3-RC	38179	58	39,3	1	NZ_WKLA01000001, complement(589070..627248)
VRA_2sq_f P2	44025	61	37,9	0	NZ_WKLA01000001, 108148..152172
WDN19 P1	38777	62	39,8	1 (1)	NZ_AP024685, 39849..78625
WDN19 P2	35440	54	40,0	1	NZ_AP024685, 1804328..1839767
WDN19 P3	44080	63	39,0	0	NZ_AP024685, join(1946333..1967462,1..22950)
WiKim38 P1	44076	65	39,3	0	NZ_CP017124, 658472..702547
WiKim38 P2-RC	29340	43	38,2	0 (1)	NZ_CP017124, complement(1522489..1551828)
ZJUNIT8 P1	35456	43	41,8	1 (1)	NZ_CP029966, 542769..578224
ZJUNIT8 P2-RC	32754	51	41,9	0 (1)	NZ_CP029966, complement(1295227..1327980)

*1: RC (reverse complement) was listed for proper prophage alignment. Prophage starts (if present/sequenced) with an integrase and ends with genes for host lysis.

*2: Joined sequences: Prophage genome might contain missing sequence parts due to non-complete sequencing data. Prophage was located on multiple contigs.

Supplementary Table 5. Methylase/methyltransferase genes annotated in *L. curvatus* prophages and whether restriction enzymes (with their respective locus tag) were located directly next to them, as well as the predicted methylation type according to rebase.neb.org.

L. curvatus phage	Annotated product name	Methylase/ Methyltransferase locus tag	Restriction enzyme present? (locus tag)	Methylation according to BLAST at rebase.neb.com
DSM20019 P1	DNA cytosine methyltransferase	LCU_RS02350	no	C5
DSM20019 P2	DNA cytosine methyltransferase	LCU_RS03890	no	C5
ELA214117 P1	DNA cytosine methyltransferase	LOX61_RS00330	no	C5
ELA214388 P1	DNA adenine methylase	LBW12_RS02565	no	n.d.
FAM25164 P1	BREX-1 system adenine-specific DNA-methyltransferase PglX	JYG89_RS01120	integrase (tyrosine recombinase) (JYG89_RS01125)	N6A
HFS9 P1	DNA (cytosine-5-)-methyltransferase	NB814_RS00990	no	C5 **
MRS6 P1	DNA cytosine methyltransferase	CG419_RS03870	HNH homing endonuclease (CG419_RS03875)	C5
NRIC0822 P1	DNA (cytosine-5-)-methyltransferase	OA78_RS03005	no	C5
TMW 1.2270 P1	DNA adenine methylase	KNO48_01670	no	n.d.
TMW 1.591 P1	phage N-6-adenine-methyltransferase	KNP41_00790	integrase (tyrosine recombinase) (KNP41_00775)	n.d.
TMW 1.591 P1	SAM-dependent DNA methyltransferase	KNP41_00780	no	N6A **
TMW 1.706 P1	DNA adenine methyltransferase	KNP59_03110	no	N6A **
TMW 1.706 P1	Cytosine-specific methyltransferase	KNP59_03060	no	C5
TMW 1.706 P2	Cytosine-specific methyltransferase	KNP59_05685	no	C5
VRA_2sq_f P2	DNA modification methylase	GKC32_RS00630	no	n.d.
WiKim38 P1	class I SAM-dependent methyltransferase	LCW_RS03455	no	n.d.
TMW 1.595 P1	phage N-6-adenine-methyltransferase	A4W80_04775	no	n.d.
TMW 1.1390 P2	site-specific DNA-methyltransferase (adenine-specific)	A4W74_07330	no	n.d.

*n.d.: Not determinable.
**: Poor alignment quality.

The following lactobacilli phage genomes (sorted after infected host species), from which the integrase genes, used for the construction of phylogenetic trees, were obtained, can be accessed under their respective accession number at the NCBI website:

Levilactobacillus brevis: ATCCB (MK504445.1), JNU P2 (MN830254), JNU P4 (MN830255), Lb (MG020111.1), LBR48 (GU967410.1), SA-C12 (KU052488.1). *Lacticaseibacillus casei*: A2 (NC_004112.1), J-1 (KC171646.1), LJ (MF999224.1), phiAT3 (NC_005893.1), PL-1 (KC171647.1), PLE2 (KU848187.1), PLE3 (KU848186.1). *Lactobacillus delbrueckii*: JCL1032 (EU409559.1), Ldl1 (KM514685.1), LL-H (EF455602.1), phiJB (KF188409.1). *Limosilactobacillus fermentum*: JNU P1 (MN830252), JNU P5 (MN830253), LF1 (HQ141410.1), phiPYB5 (GU323708.1). *Lactobacillus gasseri*: JNU P11 (MN830257), JNU P7 (MN830256), KC5a (DQ320509.1), phi jlb1 (KF767351.1), phiadh (AJ131519.1). *Lactobacillus jensenii*: Lv-1 (EU871039.1). *Ligilactobacillus murinus*: phiEF-1.1 (MF041990.1). *Lacticaseibacillus paracasei*: CL2 (KR905067.1), iLp84 (KR905069.1), JNU P10 (MN830259), JNU P9 (MN830258), T25 (AP018361.1). *Lactiplantibacillus plantarum*:

ATCC 8014-B2 (JX486088.1), P1 (KX223815.1), P2 (KY381600.1), phig1e (NC_004305.1), Sha1 (HQ141411.1). *Limosilactobacillus reuteri*: LR1 (MH837542.1), LR2 (MH837543.1). *Lacticaseibacillus rhamnosus*: Lrm1 (EU246945.1). *Fructilactobacillus sanfranciscensis*: EV3 (LN885237.1).

Following ***L. sakei*** genomes harbouring prophages (indicated with "P" and a number; see round brackets) with integrase genes (Locus tags of integrase genes after genome accession number in brackets) were used in the phylogenetic tree analysis and can be accessed under their respective accession number at the NCBI website:

C21B (NZ_CP043730; P1: FXV74_RS01725), CBA3635 (NZ_CP059697; P1: H3M14_RS01445; P2: H3M14_RS08730), DS4 (NZ_CP025839; P1: C0213_RS03135), E28G (NZ_CP043728; P1: FX990_RS01970), FAM18311 (NZ_CP020459; P1: B4V05_RS06780), J54 (NZ_LT960790; P1: LSAJ54_RS03065), J64 (NZ_LT960781; P1: LSAJ64_RS03270; P2: LSAJ64_RS04575; P3: LSAJ64_RS06930), J112 (NZ_LT907933; P1: LSAJ112_RS03880), LK-145 (NZ_AP017931; P1: CCX78_RS02340), LZ217 (NZ_CP032652; P1: CFK76_RS05015), MFPB16A1401 (NZ_LT960788; P1: MFPB16_RS03215), MFPB19 (NZ_LT960784; P1: MFPB19_RS03355), ob4.1 (NZ_CP075489; P1: KIK01_RS00645; P2: KIK01_RS07945), Probio65 (NZ_CP020806; P1: LP065_RS09965; P2: LP065_RS10215), TMW 1.23 (JAHIAK000000000; P1: KNO63_08630), TMW 1.46 (CP015487; P1: A4W82_04315; P2: A4W82_06310), TMW 1.1290 (JAHIAJ000000000; P1: KNO49_00275), TMW 1.1386 (JAHIAI000000000; P1: KNO52_05830), TMW 1.1393 (JAHIAH000000000; P1: KNO57_04745), TMW 1.1397 (JAMOWE000000000; P1: NCX38_RS02530), TMW 1.1398 (CP017275; P1: A4W88_04030), WiKim0063 (NZ_CP022709; P1: LBS_RS02010), WiKim0072 (NZ_CP025136; P1: CW750_RS08285).

Following ***L. curvatus*** phage integrases (locus tags are listed in round brackets; accession numbers of genomes are listed in the main manuscript), were used in the phylogenetic tree analysis:

CBA3617 P1 (FGL79_RS00125), CBA3617 P2 (FGL79_RS09095), DRD-164 P1 (NE283_RS06020), DRD-164 P2 (NE283_RS08620), DSM20019 P1 (LCU_RS02270), DSM20019 P2 (LCU_RS03990), ELA214061 P1 (LOX59_RS00455), ELA214117 P1 (LOX61_RS00430), ELA214117 P2 (LOX61_RS01320), ELA214388 P1 (LBW12_RS02490), ELA214388 P2 (LBW12_RS05470), FAM25164 P1 (JYG89_RS00995), FLEC03 P1 (LCUFL03_RS07090), HFS9 P1 (NB814_RS01095), IRG2 P1 (CYK59_RS03755), KG6 P1 (CGZ47_RS06090), MRS6 P1 (CG419_RS03720), NFH-Km12 P1 (NFHkm12_15840), NRIC0822 P1 (OA78_RS02885), RI-406 P1 (B0D99_RS01065), SRCM103465 P1 (EQK21_RS03860), TMW 1.27 P1 (A4W75_04745), TMW 1.27 P2 (A4W75_05465), TMW 1.591 P1 (KNP41_00860), TMW 1.595 P1 (A4W80_04700), TMW 1.624 P1 (A4W72_02990), TMW 1.624 P2 (A4W72_06155), TMW 1.706 P1 (KNP59_03165), TMW 1.706 P2 (KNP59_05605), TMW 1.1365 P1 (KNP66_00070), TMW 1.1365 P2 (KNP66_03445), TMW 1.1365 P3 (KNP66_05285), TMW 1.1381 P1 (A4W73_07165), TMW 1.1390 P1 (A4W74_06760), TMW 1.1390 P2 (A4W74_07400), TMW 1.1408 P1 (KNO77_02360), TMW 1.1928 P1 (DT351_RS03185), TMW 1.1928 P2 (DT351_RS07510), TMW 1.2270 P1 (KNO48_01600), TMW 1.2272 P1 (KNP65_06745), VRA_2sq_f P1 = P3 (GKC32_RS03210), VRA_2sq_f P2 (GKC32_RS00480), WDN19 P1 (LTWDN19_RS00250), WDN19 P2 (LTWDN19_RS09300), WDN19 P3 (LTWDN19_RS10100), WiKim38 P1 (LCW_RS03355), WiKim38 P2 (LCW_RS07945), ZJUNIT8 P1 (C0W45_RS02830), ZJUNIT8 P2 (C0W45_RS06960).

	#	1	2	3	4	5	6	7	8	9	10	11	12	13	14	15	16	17
Latilactobacillus_curvatus_phage_CRA3617_P1	1		95.48	85.83	88.30	87.71	86.76	87.34	90.79	0.00	0.00	0.00	87.96	86.92	90.67	90.84	0.00	90.30
Latilactobacillus_curvatus_phage_CRA3617_P2-RC	2	6.49		92.57	78.96	90.06	92.53	91.33	90.49	0.00	0.00	0.00	0.00	90.50	89.03	89.51	91.41	0.00
Latilactobacillus_curvatus_phage_DRD-164_P1	3	1.40	11.48		0.00	0.00	92.03	88.67	88.90	0.00	0.00	0.00	0.00	90.62	87.06	90.79	95.91	0.00
Latilactobacillus_curvatus_phage_DRD-164_P2-RC	4	33.65	3.16	0.47		89.17	0.00	90.32	91.11	0.00	0.00	0.00	91.41	84.64	90.21	91.66	97.79	63.10
Latilactobacillus_curvatus_phage_DSM20019_P1	5	39.65	3.00	0.46	37.43		94.17	90.04	89.93	0.00	0.00	0.00	91.22	89.56	87.96	89.59	0.00	63.74
Latilactobacillus_curvatus_phage_DSM20019_P2-RC	6	1.34	24.75	46.31	0.00	2.89		91.21	89.11	0.00	0.00	0.00	90.87	88.36	90.71	91.12	91.41	68.15
Latilactobacillus_curvatus_phage_ELA214081_P1-RC	7	5.39	4.67	2.17	17.60	11.05	4.06		92.69	0.00	0.00	0.00	90.44	86.85	96.23	89.96	0.00	63.55
Latilactobacillus_curvatus_phage_ELA214117_P1-RC	8	6.34	2.10	15.72	15.61	9.36	15.22	23.17		0.00	0.00	0.00	92.09	88.94	91.01	92.91	89.18	0.00
Latilactobacillus_curvatus_phage_ELA214117_P2	9	0.00	0.00	0.07	0.00	0.00	0.07	0.03	0.10		87.88	0.00	0.00	0.00	0.00	0.00	0.00	0.00
Latilactobacillus_curvatus_phage_ELA214388_P1	10	0.00	0.00	0.00	0.02	0.00	0.00	0.04	0.03	3.82		86.38	0.00	0.00	0.00	0.00	0.00	0.00
Latilactobacillus_curvatus_phage_ELA214388_P2-RC	11	0.00	0.00	0.00	0.00	0.00	0.00	0.00	0.00	0.00	35.87		0.00	0.00	0.00	0.00	0.00	0.00
Latilactobacillus_curvatus_phage_FAM25164_P1	12	29.31	0.11	0.00	42.41	40.00	8.79	15.43	6.82	0.02	0.03	0.00		85.79	87.80	91.47	0.00	83.61
Latilactobacillus_curvatus_phage_FLEC03_P1-RC	13	5.43	4.86	47.32	2.08	4.95	34.15	9.09	10.11	0.07	0.00	0.00	6.64		89.38	90.57	91.73	82.35
Latilactobacillus_curvatus_phage_HFS9_P1-RC	14	1.83	1.43	1.33	8.72	6.06	1.25	45.56	22.61	0.04	0.01	0.00	12.06	1.78		91.22	0.00	0.00
Latilactobacillus_curvatus_phage_IRG2_P1	15	4.75	6.36	16.58	9.76	8.60	17.30	18.41	62.21	0.06	0.01	0.00	13.73	12.61	16.49		90.29	76.41
Latilactobacillus_curvatus_phage_KG6_P1-RC	16	0.00	10.67	67.79	1.39	0.00	48.05	0.00	15.59	0.08	0.00	0.00	0.01	46.13	0.00	16.16		0.00
Latilactobacillus_curvatus_phage_MRS6_P1	17	2.29	0.00	0.07	1.90	1.65	3.06	2.06	0.05	0.00	0.00	0.00	3.78	2.19	0.05	3.34	0.00	
Latilactobacillus_curvatus_phage_NFH-Km12_P1-RC	18	0.00	0.01	0.00	0.00	0.00	0.00	0.00	0.00	0.16	36.30	46.45	0.01	0.00	0.00	0.01	0.00	0.00
Latilactobacillus_curvatus_phage_NRIC0822_P1_joined_sequences	19	5.82	0.00	0.00	0.00	4.01	0.00	0.99	3.70	0.00	0.01	0.01	1.85	0.00	0.00	0.93	0.01	0.51
Latilactobacillus_curvatus_phage_RI-406_P1-RC	20	10.68	0.00	0.06	0.00	0.00	5.60	0.19	0.05	0.01	0.01	0.02	5.65	0.00	0.12	0.00	0.00	0.00
Latilactobacillus_curvatus_phage_SRCM103485_P1	21	0.00	0.00	0.10	0.00	0.00	0.00	0.00	0.00	0.00	0.44	0.00	0.00	0.00	0.01	0.00	0.00	3.52
Latilactobacillus_curvatus_phage_TMW_1.1385_P1	22	0.55	0.00	0.01	0.00	0.55	0.01	1.46	2.81	0.01	0.00	0.00	0.00	0.54	1.33	1.32	0.01	0.00
Latilactobacillus_curvatus_phage_TMW_1.1385_P2	23	0.00	0.00	0.00	0.00	0.00	0.00	0.00	0.00	0.03	23.80	0.01	0.00	0.00	0.00	0.00	0.00	0.00
Latilactobacillus_curvatus_phage_TMW_1.1385_P3	24	2.67	0.00	0.10	0.00	2.86	2.37	0.07	3.20	0.00	0.01	0.01	1.94	0.00	2.07	4.21	0.00	6.02
Latilactobacillus_curvatus_phage_TMW_1.1381_P1	25	5.53	0.00	0.00	0.13	3.94	0.00	0.00	3.83	0.00	0.53	0.00	0.00	0.00	0.50	3.02	0.00	0.00
Latilactobacillus_curvatus_phage_TMW_1.1408_P1	26	3.39	0.00	0.01	0.00	0.49	0.01	0.00	1.22	0.00	0.07	0.12	0.00	0.01	0.00	1.24	0.01	0.00
Latilactobacillus_curvatus_phage_TMW_1.1447_P1	27	0.01	0.00	0.00	0.00	0.00	0.00	2.69	0.77	0.83	0.02	0.02	0.01	8.12	3.05	1.11	0.00	2.93
Latilactobacillus_curvatus_phage_TMW_1.1928_P1	28	2.53	0.14	52.18	2.86	2.64	29.83	4.18	14.50	0.07	0.00	4.72	6.62	61.33	1.18	13.79	61.41	1.59
Latilactobacillus_curvatus_phage_TMW_1.1928_P2-RC	29	5.46	0.00	0.00	0.13	3.89	0.00	0.00	2.31	0.00	0.54	0.00	0.00	0.00	0.50	2.89	0.00	0.00
Latilactobacillus_curvatus_phage_TMW_1.2270_P1	30	0.00	0.00	0.00	0.00	0.72	0.00	0.00	0.00	0.00	43.92	49.00	0.00	0.00	0.00	0.00	0.00	0.00
Latilactobacillus_curvatus_phage_TMW_1.2272_P1	31	0.00	0.00	0.00	0.00	0.00	2.50	0.00	0.00	0.00	0.00	0.00	0.00	0.00	1.09	1.06	0.00	0.00
Latilactobacillus_curvatus_phage_TMW_1.591_P1	32	28.39	11.96	23.27	33.38	31.76	10.93	16.41	3.39	0.00	0.00	0.00	25.64	26.29	6.10	7.11	22.92	3.26
Latilactobacillus_curvatus_phage_TMW_1.708_P1_joined_sequences	33	1.35	25.04	46.82	0.00	2.92	97.75	3.97	15.27	0.07	0.00	0.00	8.89	34.55	1.27	17.51	48.53	3.10
Latilactobacillus_curvatus_phage_TMW_1.708_P2_joined_sequences	34	40.12	3.03	0.47	39.45	97.60	2.92	11.10	9.47	0.00	0.00	0.00	39.71	5.01	7.80	10.24	0.00	3.12
Latilactobacillus_curvatus_phage_VRA_2sq_f_P1_=_P3-RC	35	2.13	0.00	0.00	14.17	9.43	0.00	21.31	22.64	0.00	0.00	0.00	15.58	0.06	9.07	15.22	0.00	0.00
Latilactobacillus_curvatus_phage_VRA_2sq_f_P2	36	0.65	0.00	0.10	0.00	0.77	0.00	0.14	0.73	0.00	0.00	0.00	0.00	0.06	1.00	1.68	0.11	0.00
Latilactobacillus_curvatus_phage_WDN19_P1	37	2.94	26.96	7.73	0.13	6.09	21.88	2.80	4.04	0.00	0.01	0.00	0.01	3.13	2.27	6.56	7.94	0.00
Latilactobacillus_curvatus_phage_WDN19_P2	38	62.06	1.99	1.40	33.38	39.92	1.33	5.19	7.56	0.02	0.00	0.00	29.93	2.09	7.54	7.38	0.00	6.58
Latilactobacillus_curvatus_phage_WDN19_P3	39	4.74	0.00	0.11	0.00	0.00	0.00	0.96	0.05	0.00	0.01	0.01	2.14	0.00	0.00	0.00	0.00	0.51
Latilactobacillus_curvatus_phage_WiKim38_P1	40	16.88	1.91	1.40	0.00	0.00	4.54	1.95	3.29	0.00	0.01	0.01	5.93	1.98	2.71	1.64	0.00	2.52
Latilactobacillus_curvatus_phage_WiKim38_P2-RC	41	0.00	0.00	0.00	0.00	0.00	0.00	0.00	0.00	0.00	0.01	0.00	0.00	0.00	0.00	0.00	0.00	0.00
Latilactobacillus_curvatus_phage_ZJUNIT8_P1	42	0.05	0.34	13.40	0.00	0.12	12.31	3.19	0.53	0.00	0.00	0.00	2.86	17.04	0.84	0.86	20.69	0.00
Latilactobacillus_curvatus_phage_ZJUNIT8_P2-RC	43	2.27	0.00	0.00	0.00	0.00	0.00	0.00	0.00	0.00	0.50	1.61	0.00	1.29	0.00	0.01	0.00	0.00
Latilactobacillus_curvatus_phage_TMW_1.27_P1-RC	44	0.00	0.00	0.11	0.01	0.00	2.39	0.07	0.00	0.00	0.01	0.01	0.00	0.00	0.00	0.00	0.00	0.50
Latilactobacillus_curvatus_phage_TMW_1.27_P2-RC	45	0.17	5.92	15.92	2.12	4.98	8.50	4.89	0.25	0.00	0.00	0.00	8.04	18.94	0.11	3.07	16.53	0.00
Latilactobacillus_curvatus_phage_TMW_1.595_P1	46	4.16	11.04	62.25	2.16	3.50	30.17	5.79	15.23	0.07	0.00	0.00	4.53	61.68	1.87	16.80	67.36	1.65
Latilactobacillus_curvatus_phage_TMW_1.624_P1	47	2.61	0.00	0.01	0.54	3.33	0.01	0.00	0.45	0.00	3.68	1.86	0.00	0.01	0.00	1.21	0.01	0.00
Latilactobacillus_curvatus_phage_TMW_1.624_P2-RC	48	1.36	10.95	61.34	0.77	0.00	40.33	10.92	16.99	0.07	0.00	0.00	2.76	48.44	2.44	19.11	69.12	0.00
Latilactobacillus_curvatus_phage_TMW_1.1390_P1-RC	49	39.32	0.11	0.03	50.67	52.85	0.01	22.81	12.84	0.02	0.01	0.47	41.88	3.19	13.27	12.24	0.00	2.67
Latilactobacillus_curvatus_phage_TMW_1.1390_P2-RC	50	0.64	0.00	0.00	0.05	0.01	0.00	0.00	0.70	2.70	6.98	0.32	0.01	0.00	0.00	0.60	0.00	0.00

#	18	19	20	21	22	23	24	25	26	27	28	29	30	31	32	33	34
1	0.00	93.10	93.49	0.00	0.00	0.00	91.54	90.30	85.06	0.00	63.65	90.35	0.00	0.00	86.99	88.76	87.71
2	0.00	0.00	0.00	0.00	0.00	0.00	0.00	0.00	0.00	0.00	0.00	0.00	0.00	0.00	92.05	92.53	90.06
3	0.00	0.00	0.00	0.00	0.00	0.00	0.00	0.00	0.00	0.00	93.15	0.00	0.00	0.00	91.66	92.03	0.00
4	0.00	0.00	0.00	0.00	0.00	0.00	0.00	0.00	0.00	0.00	68.45	0.00	0.00	0.00	86.57	0.00	89.10
5	0.00	89.99	0.00	0.00	0.00	0.00	88.34	89.28	0.00	0.00	65.86	89.26	0.00	0.00	87.95	94.17	100.00
6	0.00	0.00	86.94	0.00	0.00	0.00	86.72	0.00	0.00	0.00	91.30	0.00	0.00	81.10	93.68	100.00	94.17
7	0.00	0.00	0.00	0.00	90.22	0.00	0.00	0.00	0.00	90.88	88.04	0.00	0.00	0.00	92.37	91.59	90.04
8	0.00	97.67	0.00	0.00	97.12	0.00	88.71	91.16	87.39	0.00	69.61	86.02	0.00	0.00	87.56	89.17	89.93
9	0.00	0.00	0.00	0.00	0.00	0.00	0.00	0.00	0.00	0.00	0.00	0.00	0.00	0.00	0.00	0.00	0.00
10	87.43	0.00	0.00	0.00	0.00	90.11	0.00	0.00	0.00	0.00	0.00	0.00	88.57	0.00	0.00	0.00	0.00
11	89.77	0.00	0.00	0.00	0.00	0.00	0.00	0.00	0.00	0.00	88.42	0.00	90.14	0.00	0.00	0.00	0.00
12	0.00	76.28	86.56	0.00	0.00	0.00	81.51	0.00	0.00	0.00	83.57	0.00	0.00	0.00	89.67	90.87	91.63
13	0.00	0.00	0.00	0.00	0.00	0.00	0.00	0.00	0.00	89.44	92.29	0.00	0.00	0.00	92.41	88.36	89.56
14	0.00	0.00	0.00	0.00	92.37	0.00	87.41	0.00	0.00	85.27	93.41	0.00	0.00	81.86	84.58	90.71	88.06
15	0.00	0.00	0.00	0.00	90.81	0.00	88.98	91.30	87.46	83.97	91.64	91.33	0.00	84.13	88.72	91.12	89.11
16	0.00	0.00	0.00	0.00	0.00	0.00	0.00	0.00	0.00	0.00	94.69	0.00	0.00	0.00	90.09	91.41	0.00
17	0.00	0.00	0.00	84.04	0.00	0.00	86.47	0.00	0.00	85.05	84.64	0.00	0.00	0.00	81.36	88.15	85.15
18		0.00	0.00	0.00	0.00	86.03	0.00	0.00	85.06	0.00	0.00	0.00	94.00	0.00	0.00	0.00	0.00
19	0.00		95.01	86.84	0.00	0.00	93.75	0.00	87.97	93.43	0.00	0.00	0.00	91.16	0.00	0.00	89.99
20	0.00	66.46		86.27	0.00	0.00	95.46	0.00	0.00	95.00	0.00	0.00	0.00	89.64	0.00	86.94	0.00
21	0.00	36.92	36.97		0.00	0.00	87.56	83.57	0.00	89.26	0.00	83.37	0.00	85.26	0.00	0.00	0.00
22	0.00	0.00	0.00	0.00		82.57	92.50	91.64	94.84	91.82	92.08	89.74	0.00	0.00	89.80	0.00	0.00
23	3.46	0.00	0.00	0.00	6.90		0.00	0.00	0.00	0.00	0.00	0.00	81.44	0.00	0.00	0.00	0.00
24	0.00	51.89	51.30	41.35	1.20	0.00		93.06	0.00	96.11	0.00	93.06	0.00	89.21	0.00	86.72	88.34
25	0.00	0.46	0.03	2.45	51.52	0.00	2.21		93.97	0.00	0.00	95.17	0.00	0.00	0.00	0.00	89.26
26	1.14	1.05	0.00	0.00	82.56	0.25	0.44	49.01		0.00	0.00	91.83	0.00	0.00	0.00	0.00	0.00
27	0.01	60.47	46.84	39.43	1.30	0.00	62.91	0.76	0.83		86.24	0.00	0.00	91.83	91.62	0.00	0.00
28	0.01	0.57	0.00	0.00	1.33	0.00	0.56	0.87	0.66	2.45		0.00	0.00	0.00	86.60	91.30	85.88
29	0.01	0.15	0.03	2.42	10.92	0.05	2.17	69.61	48.41	0.75	0.66		0.00	0.00	0.00	0.00	89.28
30	59.87	0.00	0.02	0.00	0.00	4.78	0.64	0.00	0.75	0.01	0.61	0.00		0.00	0.00	0.00	0.00
31	0.00	4.89	6.90	8.83	0.00	0.00	16.62	0.00	0.03	11.47	0.59	0.00	0.00		0.00	81.10	0.00
32	0.00	0.94	0.25	0.00	1.49	0.00	0.00	0.00	0.00	2.41	19.10	0.00	0.00	0.00		93.68	88.75
33	0.00	0.00	5.68	0.00	0.01	0.00	2.40	0.00	0.01	0.00	30.17	0.00	0.00	2.53	11.06		94.17
34	0.00	4.06	0.00	0.00	0.55	0.00	2.89	3.98	0.50	0.00	2.67	3.93	0.73	0.00	31.37	2.96	
35	1.00	0.79	0.00	0.00	0.00	0.00	0.72	0.76	2.93	0.04	0.61	0.76	0.00	0.00	0.06	0.00	9.54
36	0.00	42.43	35.72	39.58	1.92	0.02	41.36	0.72	0.00	48.41	0.67	0.71	0.00	15.07	0.06	0.00	0.79
37	0.01	0.97	0.00	0.00	1.29	0.00	3.15	2.37	1.11	0.01	6.07	2.34	0.01	0.57	9.95	22.14	6.17
38	0.00	1.02	0.00	0.00	1.38	0.00	3.31	4.75	2.73	0.81	2.53	4.69	0.00	0.00	30.45	1.35	39.61
39	0.00	55.01	52.83	37.67	0.00	0.00	51.82	1.87	0.00	49.06	0.00	1.85	0.00	4.74	0.92	0.00	0.00
40	0.00	52.45	53.72	31.98	0.00	0.00	46.48	0.00	0.00	51.04	0.00	0.00	0.00	7.56	1.94	4.59	0.00
41	6.35	0.00	0.00	0.00	0.00	3.28	0.00	2.71	6.15	0.00	0.71	2.89	0.81	2.44	0.00	0.00	0.00
42	0.00	2.81	3.37	0.00	0.09	0.00	4.01	0.00	0.00	1.66	23.08	0.01	0.00	2.89	12.25	11.65	0.12
43	0.01	0.00	0.00	0.00	52.78	0.05	4.00	64.33	60.76	0.00	0.00	64.18	1.49	0.00	0.00	0.00	0.00
44	0.00	44.64	45.76	41.93	0.00	0.00	64.00	1.86	0.00	58.30	0.00	1.53	0.00	2.23	0.00	2.42	0.00
45	0.00	0.00	3.71	0.00	0.08	0.00	2.97	0.00	0.00	0.15	16.26	0.01	0.00	0.00	20.36	7.81	5.04
46	0.00	0.00	0.00	0.00	0.57	0.00	0.01	0.78	0.01	2.72	66.44	0.77	0.00	0.00	28.11	39.60	3.54
47	1.08	0.43	0.03	2.28	58.66	0.00	0.42	56.28	59.58	0.79	2.53	49.64	2.05	0.00	0.00	0.01	3.38
48	0.00	0.86	0.00	0.00	1.53	0.00	0.00	0.00	0.01	3.34	61.88	0.00	0.00	0.00	32.17	40.79	0.00
49	0.00	0.23	0.41	0.00	0.54	0.00	0.67	1.19	0.00	0.48	2.35	1.16	0.46	0.00	31.60	0.01	66.22
50	0.00	0.57	0.00	0.00	2.06	52.40	0.00	5.59	3.07	0.80	0.62	5.52	6.18	0.00	0.00	0.00	0.01

#	35	36	37	38	39	40	41	42	43	44	45	46	47	48	49	50
1	80.95	0.00	96.04	94.61	67.88	94.07	0.00	0.00	84.68	0.00	0.00	87.39	83.65	87.47	86.02	0.00
2	0.00	0.00	95.26	90.54	0.00	87.23	0.00	0.00	0.00	0.00	81.32	91.91	0.00	91.50	0.00	0.00
3	0.00	0.00	93.44	86.09	0.00	85.93	0.00	90.09	0.00	0.00	89.22	95.83	0.00	93.56	0.00	0.00
4	86.26	0.00	0.00	86.71	0.00	0.00	0.00	0.00	0.00	0.00	86.56	85.23	0.00	0.00	91.36	0.00
5	84.04	0.00	89.88	86.14	0.00	0.00	0.00	0.00	0.00	0.00	88.51	88.01	83.13	0.00	91.43	0.00
6	0.00	0.00	93.60	86.56	0.00	88.99	0.00	85.22	0.00	92.77	81.36	91.67	0.00	91.44	0.00	0.00
7	88.73	0.00	92.96	91.37	0.00	89.37	0.00	96.68	0.00	0.00	88.34	82.00	0.00	90.21	89.94	0.00
8	88.31	0.00	89.26	91.78	0.00	91.36	0.00	0.00	0.00	0.00	0.00	89.05	0.00	89.06	89.55	0.00
9	0.00	0.00	0.00	0.00	0.00	0.00	0.00	0.00	0.00	0.00	0.00	0.00	0.00	0.00	0.00	85.97
10	0.00	0.00	0.00	0.00	0.00	0.00	0.00	0.00	0.00	0.00	0.00	0.00	98.69	0.00	0.00	85.02
11	0.00	0.00	0.00	0.00	0.00	0.00	0.00	0.00	85.31	0.00	0.00	0.00	99.86	0.00	0.00	0.00
12	86.72	0.00	0.00	86.18	80.41	87.86	0.00	88.38	0.00	0.00	86.09	83.26	0.00	91.71	91.10	0.00
13	0.00	0.00	91.49	91.57	0.00	89.36	0.00	91.32	82.00	0.00	88.44	91.52	0.00	90.90	86.48	0.00
14	86.03	0.00	93.31	86.49	0.00	89.76	0.00	0.00	0.00	0.00	0.00	88.92	0.00	91.29	88.26	0.00
15	87.89	82.55	93.42	86.69	0.00	88.12	0.00	0.00	0.00	0.00	88.38	90.71	92.00	90.89	88.49	0.00
16	0.00	0.00	91.04	0.00	0.00	0.00	0.00	93.37	0.00	0.00	89.20	95.04	0.00	94.93	0.00	0.00
17	0.00	0.00	0.00	90.57	0.00	89.54	0.00	0.00	0.00	0.00	0.00	90.10	0.00	0.00	71.75	0.00
18	82.74	0.00	0.00	0.00	0.00	0.00	95.22	0.00	0.00	0.00	0.00	0.00	83.50	0.00	0.00	0.00
19	0.00	87.70	0.00	97.74	93.55	93.95	0.00	82.42	0.00	92.71	0.00	0.00	0.00	0.00	0.00	0.00
20	0.00	87.69	0.00	0.00	94.14	94.56	0.00	81.26	0.00	93.68	80.88	0.00	0.00	0.00	0.00	0.00
21	0.00	87.26	0.00	0.00	68.31	88.56	0.00	0.00	0.00	87.55	0.00	0.00	83.71	0.00	0.00	0.00
22	0.00	81.53	88.64	86.55	0.00	0.00	0.00	0.00	92.62	0.00	0.00	0.00	93.91	86.91	0.00	82.47
23	0.00	0.00	0.00	0.00	0.00	0.00	84.54	0.00	0.00	0.00	0.00	0.00	0.00	0.00	0.00	90.47
24	0.00	87.98	90.79	90.71	95.08	94.32	0.00	83.42	86.46	95.08	82.74	0.00	0.00	0.00	0.00	0.00
25	0.00	0.00	91.99	90.10	84.24	0.00	83.75	0.00	95.39	84.24	0.00	0.00	95.44	0.00	94.63	92.26
26	88.00	0.00	88.50	87.13	0.00	0.00	95.37	0.00	93.36	0.00	0.00	0.00	94.43	0.00	0.00	92.77
27	0.00	87.94	0.00	0.00	94.85	92.77	0.00	91.94	0.00	92.37	0.00	78.01	0.00	78.96	0.00	0.00
28	0.00	0.00	80.07	82.76	0.00	0.00	0.00	92.11	0.00	0.00	90.85	93.88	95.46	92.81	88.23	0.00
29	0.00	0.00	92.00	90.16	84.29	0.00	83.81	0.00	95.12	84.24	0.00	0.00	92.89	0.00	91.63	94.18
30	0.00	0.00	0.00	0.00	0.00	0.00	0.00	0.00	89.37	0.00	0.00	0.00	85.06	0.00	0.00	83.30
31	0.00	88.23	0.00	0.00	82.86	86.41	87.06	81.86	0.00	90.92	0.00	0.00	0.00	0.00	0.00	0.00
32	0.00	0.00	91.18	88.70	0.00	88.90	0.00	90.07	0.00	0.00	88.25	90.52	0.00	91.47	88.64	0.00
33	0.00	0.00	93.60	86.56	0.00	88.99	0.00	85.34	0.00	92.77	81.16	91.87	0.00	91.44	0.00	0.00
34	84.04	0.00	89.88	86.34	0.00	0.00	0.00	0.00	0.00	0.00	88.51	88.01	83.13	0.00	91.46	0.00
35		89.71	87.80	0.00	0.00	0.00	83.65	0.00	0.00	0.00	0.00	0.00	91.38	0.00	86.37	69.85
36	3.21		0.00	0.00	67.04	86.21	0.00	0.00	0.00	67.47	0.00	0.00	0.00	0.00	0.00	0.00
37	29.49	0.55		96.35	0.00	0.00	0.00	0.00	0.00	0.00	88.16	92.82	0.00	92.39	0.00	0.00
38	0.63	0.65	14.90		0.00	92.20	0.00	0.00	85.06	0.00	0.00	87.11	84.17	87.06	87.44	0.00
39	0.00	34.87	0.00	0.00		94.36	0.00	86.17	0.00	98.08	83.20	0.00	84.14	0.00	0.00	0.00
40	0.00	35.99	0.00	11.12	54.24		0.00	86.32	0.00	94.44	85.99	87.66	0.00	91.86	0.00	0.00
41	3.45	0.00	0.00	0.00	0.00	0.00		0.00	85.69	0.00	0.00	0.00	88.18	0.00	0.00	93.99
42	0.00	0.00	0.79	0.66	5.61	2.71	0.00		0.00	88.69	91.78	91.15	0.00	93.47	0.00	0.00
43	0.00	0.00	0.00	2.26	0.01	0.00	2.70	0.01		0.00	0.00	0.00	91.06	0.00	0.00	90.93
44	0.00	30.93	0.00	0.00	63.86	42.90	0.00	5.00	0.00		0.00	0.00	84.10	0.00	0.00	0.00
45	0.00	0.00	5.73	0.17	5.26	2.61	0.00	50.69	0.01	0.00		88.41	0.00	89.24	0.00	0.00
46	0.00	0.10	7.36	3.35	0.00	1.27	0.00	13.42	0.78	0.00	20.31		0.00	93.75	90.42	0.00
47	1.76	0.00	0.46	2.60	1.74	0.00	3.45	0.08	52.61	1.72	0.07	0.01		0.00	0.00	94.07
48	0.00	0.00	7.07	1.36	0.94	2.96	0.00	21.31	0.00	0.00	14.79	67.49	0.01		0.00	0.00
49	14.65	0.49	0.48	41.99	0.00	0.11	0.00	0.00	0.50	0.00	0.00	3.16	0.62	0.50		0.00
50	3.81	0.76	0.60	0.01	0.00	0.01	3.22	0.00	5.64	0.00	0.00	0.00	10.10	0.00	0.01	

Supplementary Figure 1. Average nucleotide consistency (pairwise comparison) of all as intact predicted ***L. curvatus*** prophages, based on a whole genome alignment (default parameters) using CLC Main workbench. For construction of the table, default parameters were chosen (Parameters: Minimum similarity fraction: 0.8; Minimum length fraction: 0.8). Lower part of the table: ANI values in percent. Upper part of the table: Aligned percentage in percent. Red filling: Higher nucleotide consistency. Blue filling: Lower nucleotide consistency.

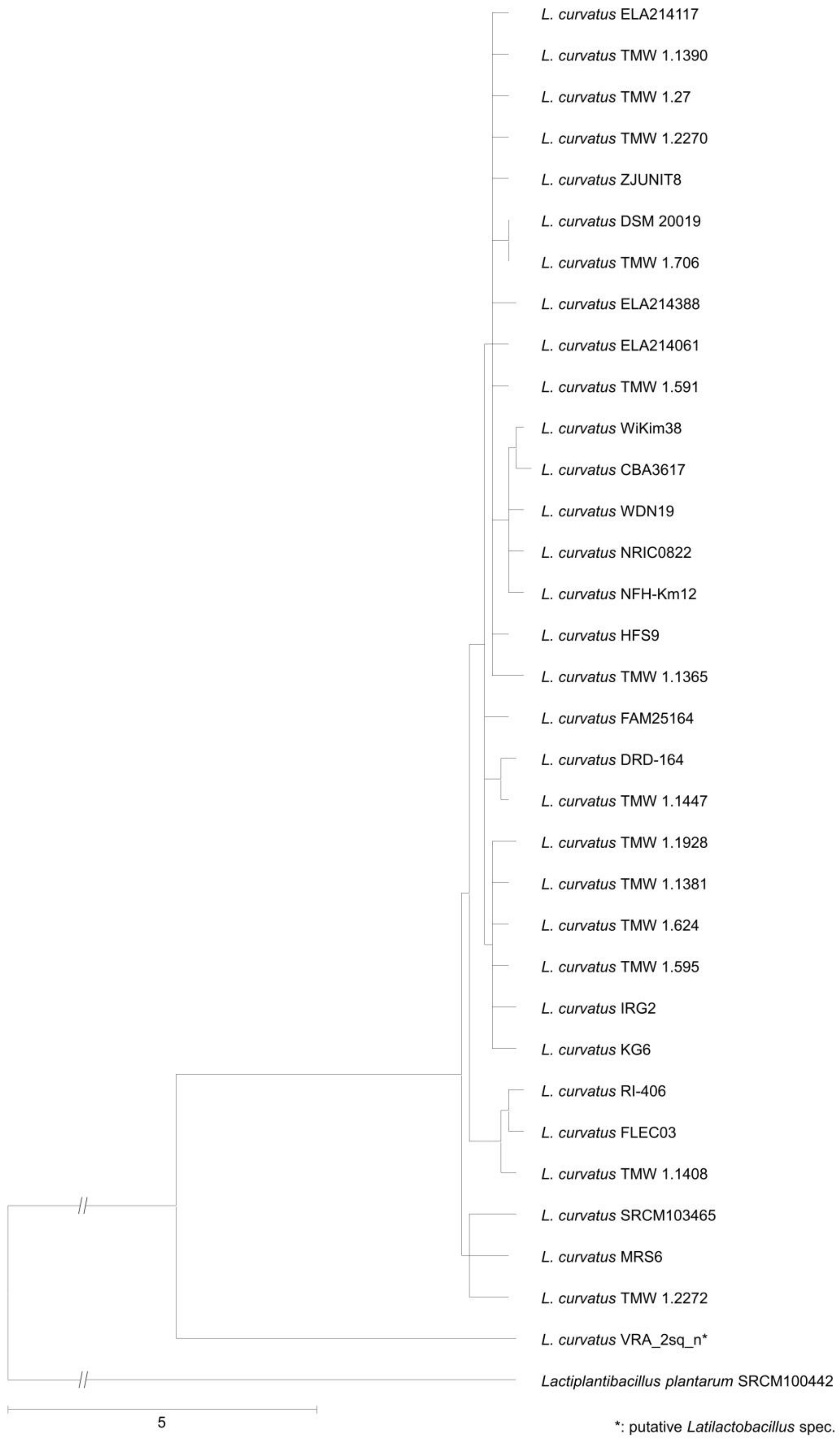

Supplementary Figure 2. Phylogenetic relationship between *L. curvatus* lysogens (strains harbouring at least one as intact predicted prophage) displayed as ANI tree created by using the neighbor joining method. The tree is based on a whole genome alignment (default parameters) using CLC Main workbench. As outgroup the genome of *Lactiplantibacillus plantarum* SRCM100442 was used.

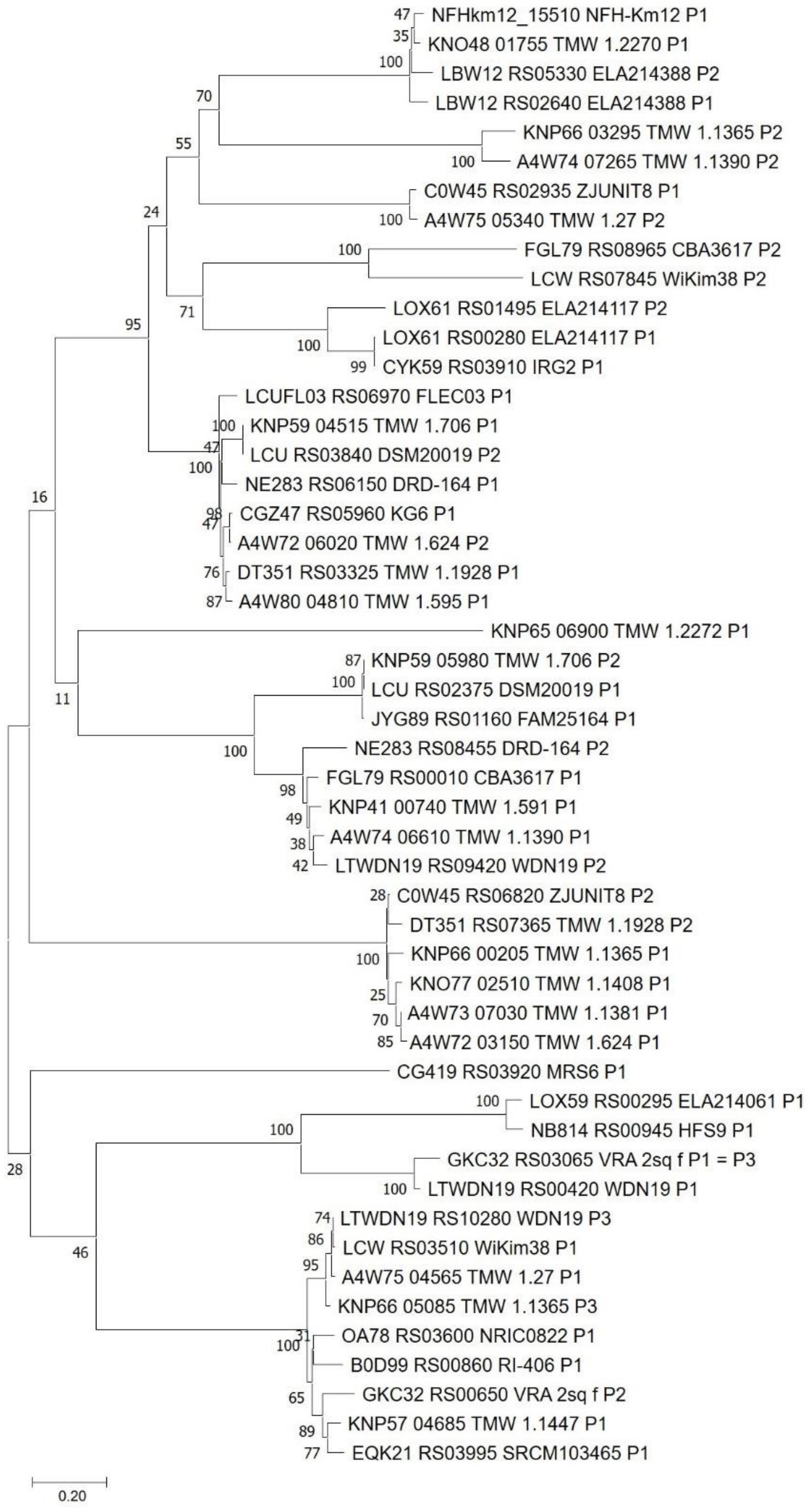

Supplementary Figure 3. Phylogenetic tree after the neighbor joining method (distance measure: Maximum Composite Likelihood method) of phage Terminase (large subunit) genes of intact *L. curvatus* phages. 1000 replicates were used in the bootstrap analysis (Jukes-Cantor model).

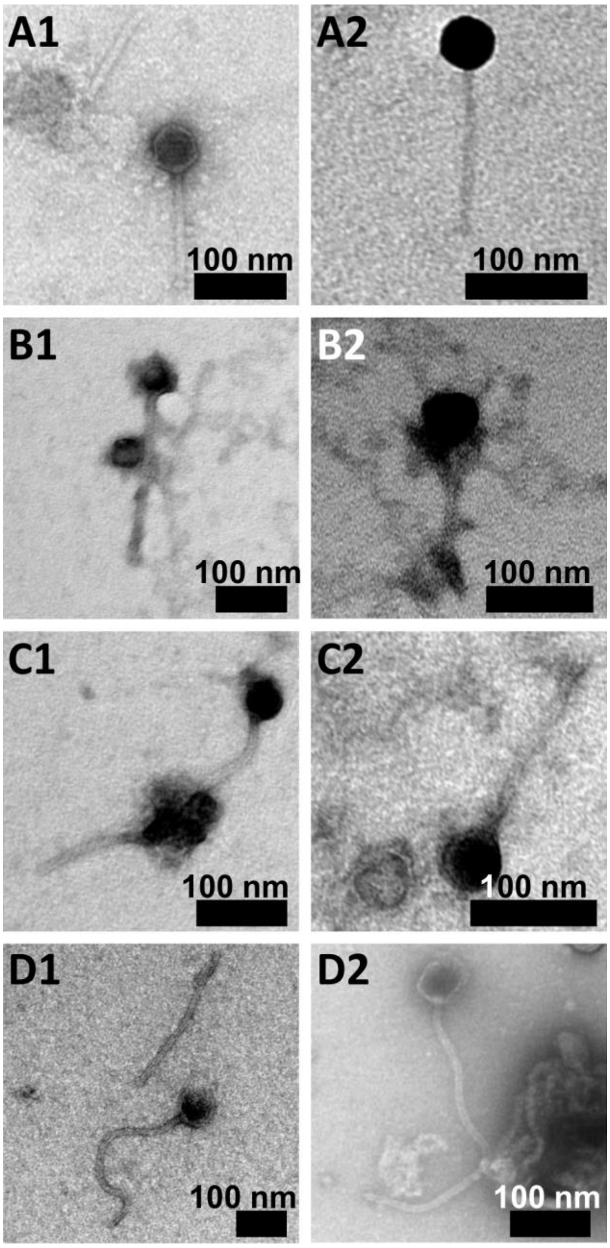

Supplementary Figure 4. Transmission electron microscopy (TEM) derived micrographs of purified post-induction lysates of different *L. curvatus* strains. For negative staining of the samples, 2% uranyl acetate was used. A1-A2: *L. curvatus* TMW 1.591. B1-B2: *L. curvatus* TMW 1.706. C1-C2: *L. curvatus* TMW 1.1365. D1-D2: *L. curvatus* TMW 1.2272. The bar size is 100 nm. The microscope was operated at 80 kV in zero-loss mode.

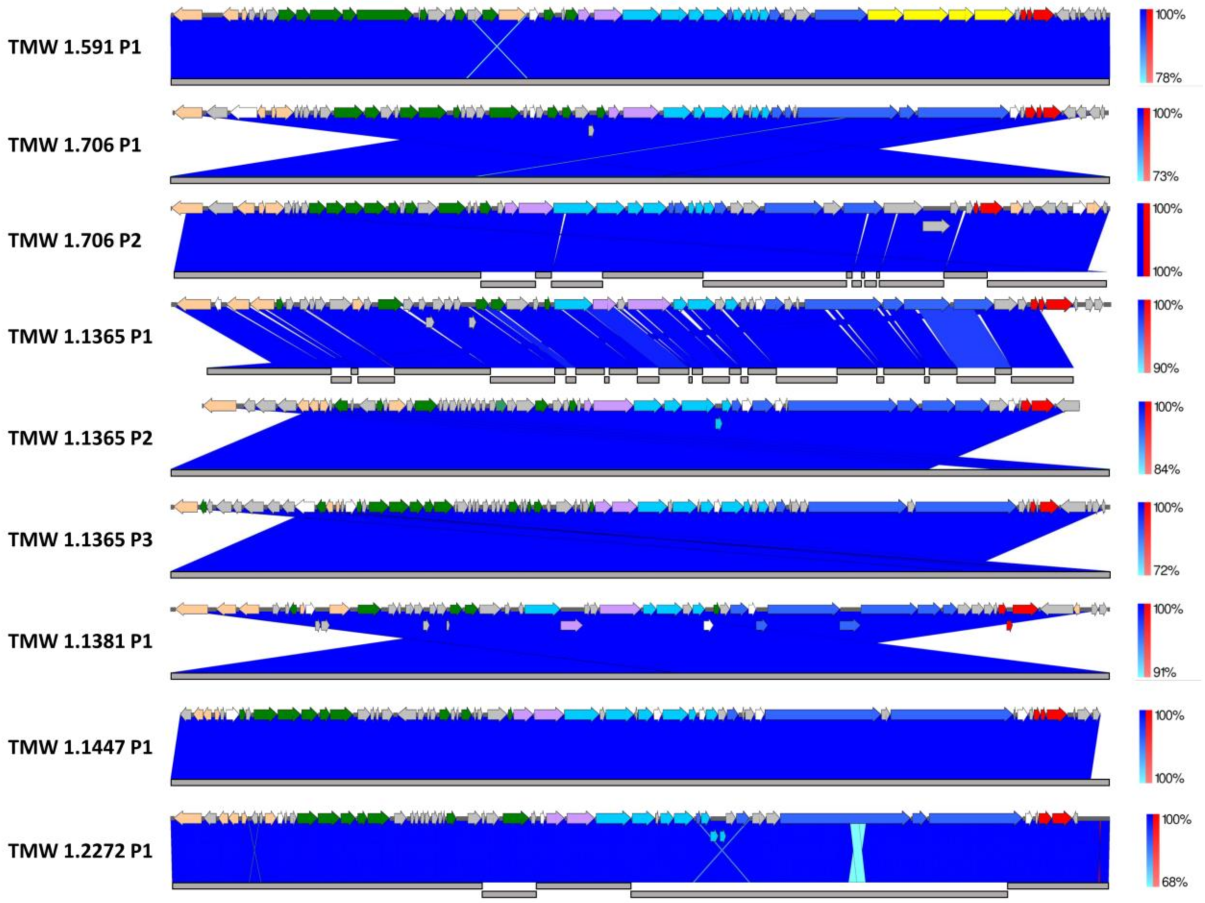

Supplementary Figure 5. BLAST analysis between each prophage and (concatenated) phage DNA isolated from purified post-induction lysates (performed via Easyfig [41]). Higher nucleotide similarities are depicted as bars (BLAST legends depicted on right side of each alignment) between (concatenated) phage contigs (grey rectangles, bottom sequences) and prophages (top sequences). Darker bars between the sequences indicate higher similarity. Prophage genes were colourised after their predicted task: Lysogeny (ochre), replication (green), packaging (lilac), head (light blue) tail (dark blue), fiber (yellow), lysis (red), hypothetical protein (grey), transposase (light yellow), unknown task (white), and tRNA (pink).

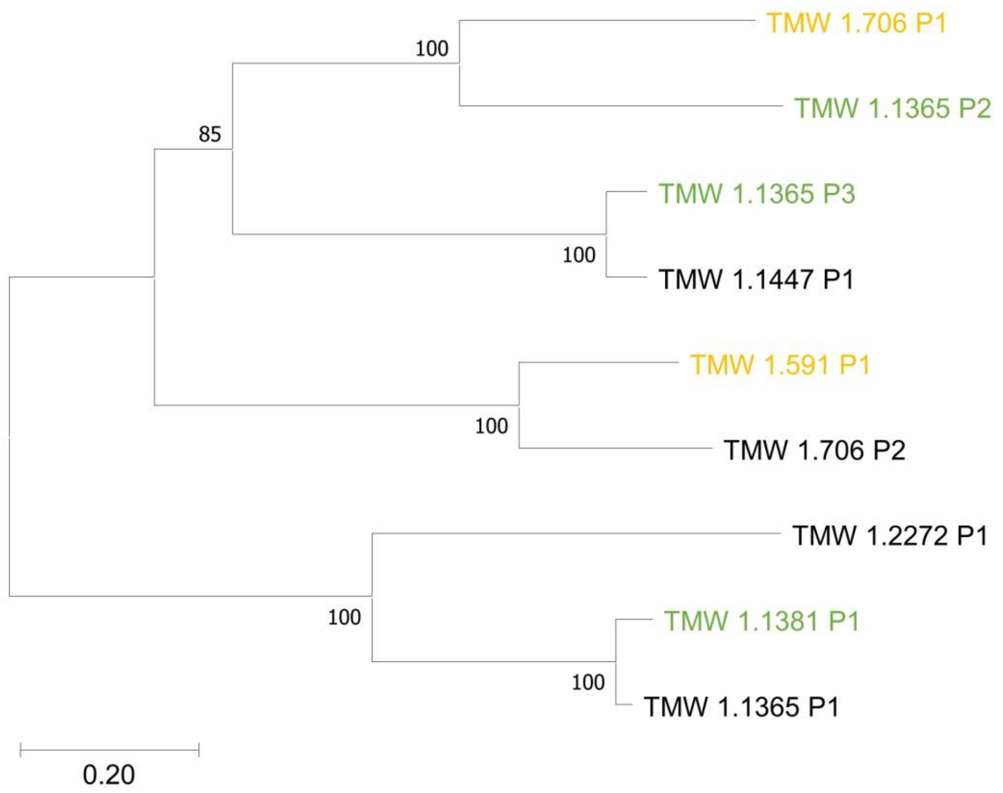

Supplementary Figure 6. Phylogenetic tree after the neighbor joining method (distance measure: Maximum Composite Likelihood method) of *L. curvatus* phage terminase gene concatemers. 1000 replicates were used in the bootstrap analysis (Jukes-Cantor model). Orange font colour indicates that a linear genome of the respective phage was retrieved after sequencing from lysates. Green colour indicates circular genomes.
